# A Normalization Circuit Underlying Coding of Spatial Attention in Primate Lateral Prefrontal Cortex

**DOI:** 10.1523/ENEURO.0301-18.2019

**Published:** 2019-04-15

**Authors:** Lyndon Duong, Matthew Leavitt, Florian Pieper, Adam Sachs, Julio Martinez-Trujillo

**Affiliations:** 1 Department of Physiology and Pharmacology, Western University, London, Ontario N6A 3K7, Canada; 2 Robarts Research Institute, London, Ontario N6A 5B7, Canada; 3 Department of Physiology, McGill University, Quebec H3A 0G4, Canada Montreal; 4 Department of Neurophysiology and Pathophysiology, University Medical Center Hamburg-Eppendorf, Hamburg 52 20246, Germany; 5 The Ottawa Hospital, University of Ottawa, Ottawa, Ontario K1H 8L6, Canada

**Keywords:** attention, macaque, neurophysiology, normalization, prefrontal cortex

## Abstract

Lateral prefrontal cortex (LPFC) neurons signal the allocation of voluntary attention; however, the neural computations underlying this function remain unknown. To investigate this, we recorded from neuronal ensembles in the LPFC of two *Macaca fascicularis* performing a visuospatial attention task. LPFC neural responses to a single stimulus were normalized when additional stimuli/distracters appeared across the visual field and were well-characterized by an averaging computation. Deploying attention toward an individual stimulus surrounded by distracters shifted neural activity from an averaging regime toward a regime similar to that when the attended stimulus was presented in isolation (winner-take-all; WTA). However, attentional modulation is both qualitatively and quantitatively dependent on a neuron’s visuospatial tuning. Our results show that during attentive vision, LPFC neuronal ensemble activity can be robustly read out by downstream areas to generate motor commands, and/or fed back into sensory areas to filter out distracter signals in favor of target signals.

## Significance Statement

Lateral prefrontal cortex (LPFC) neurons signal the allocation of voluntary attention. The neural computations underlying this phenomenon remain unknown. Here we show that neurons in the primate LPFC perform two types of normalization computations, response averaging that calibrates neural activity according to the number of stimuli in the visual field, and winner-take-all (WTA) that biases neural activity to represent attended stimuli. These computations are global, across the entire visual field, and their strength vary across neurons depending on their visuospatial tuning. We show evidence in favor of a circuit mechanism that flexibly uses normalization computations to generate a signal profile in ensembles of LPFC neurons that carries sufficient information to guide the allocation of visuospatial attention.

## Introduction

Response normalization has been reported across brain areas, sensory modalities, and species (Carandini and Heeger, 2012) and has been proposed to play a key role in cognitive functions such as attention (Boynton, 2009; Lee and Maunsell, 2009; Reynolds and Heeger, 2009; Ni et al., 2012; Verhoef and Maunsell, 2016; Ni and Maunsell, 2017). It is thought that top-down signals from executive areas containing maps of relevant objects or locations modulate normalization strength in sensory areas (Moore and Armstrong, 2003; Armstrong et al., 2009), biasing neuronal representations in favor of attended stimuli (Reynolds et al., 1999; Reynolds and Heeger, 2009). One issue that remains unclear is whether normalization also operates within executive areas of the lateral prefrontal cortex (LPFC), where neuronal responses are strongly modulated by task demands and receptive fields (RFs) often cover large parts of the visual field (Lennert and Martinez-Trujillo, 2011; Bullock et al., 2017). We hypothesize that normalization operates at large spatial scales in the LPFC, allowing the generation of saliency maps across the visual field that signal the allocation of attention.

Previous studies in visual cortices have shown that directing attention to one of multiple stimuli activating a neuron enhances the cell’s response (Moran and Desimone, 1985; Desimone and Duncan, 1995; Treue and Maunsell, 1996; Treue and Martínez Trujillo, 1999; Maunsell and Cook, 2002; Reynolds and Chelazzi, 2004; Boynton, 2009). The resultant firing rates with attention resemble those when the attended stimulus is presented alone, approximating a winner-take-all (WTA) computation that effectively filters out information from distracters (Reynolds et al., 1999; Wang, 2008; Malek et al., 2017). Some studies have elaborated on normalization models to explain these effects (Boynton, 2009; Ghose, 2009; Lee and Maunsell, 2009; Reynolds and Heeger, 2009) and demonstrated that the effects of attention can be heterogeneous depending on the location of targets and distracters within the RF excitatory (RFe) and inhibitory (RFi) regions and the size and shape of the focus of attention (Williford, 2006; Khayat et al., 2010; Niebergall et al., 2011; Verhoef and Maunsell, 2016).

Some studies have also shown that the components of normalization may be differentially tuned across neurons (Ni et al., 2012; Verhoef and Maunsell, 2016; Malek et al., 2017; Ni and Maunsell, 2017). In parietal area 7a of macaques, involved in exogenous attention (Meyers et al., 2017) normalization follows a WTA rule (Oleksiak et al., 2011). This deviates from results in other areas such as MT, where normalization follows an average (AVG) rule (Snowden et al., 1991; Recanzone et al., 1997). These discrepancies may be due to differing roles of brain areas in different types of attention (e.g., exogenous vs endogenous) and/or differences in task design and stimuli across studies. In the LPFC, where neurons encode attention signals across the entire visual field (Lennert and Martinez-Trujillo, 2011; Tremblay et al., 2015) normalization has not been thoroughly studied.

LPFC area 8a is located anterior to the frontal eye fields (FEF) on the prearcuate convexity, and posterior to area 9/46 (Petrides, 2005). Lesions to area 8a severely impair allocation of spatial attention in the contralateral visual field (Rossi et al., 2007; Pasternak et al., 2015). Neurons in this area are strongly modulated by visual stimulation and attention (Goldman-Rakic, 1995; Lennert and Martinez-Trujillo, 2011; Tremblay et al., 2015), and can be divided into contralaterally and ipsilaterally tuned relative to their hemispheric and RF center location. These tuned populations differ in the amount of suppression below their baseline firing rates when visual stimuli are shown far from their RF center (Bullock et al., 2017). The latter result suggests that normalization mechanisms may differentially operate in these two categories of LPFC neurons.

We trained two monkeys in a visuospatial attention task and recorded single cell responses from area 8a (referred here as LPFC) using multielectrode arrays. Neuronal responses to multiple stimuli were sublinear and well-described by an averaging (AVG) computation. Ipsilateral neurons were more normalized than contralateral-tuned neurons. Attending toward a neuron RFe center modulated contralateral neurons responses, shifting them from AVG to WTA. Ipsilateral neurons were less modulated and remained better explained by an AVG computation. We reduced the dimensionality of the neuronal responses to two variables representing ipsilateral and contralateral activities. Trajectories in this reduced state space converge during distracter onset and then diverge during the allocation of attention, allowing discrimination of the different experimental conditions. Finally, a linear classifier reading out the activity of all single neurons achieved quasi perfect discrimination performance using temporal windows as short as 25 ms.

## Materials and Methods

**Figure 1.**
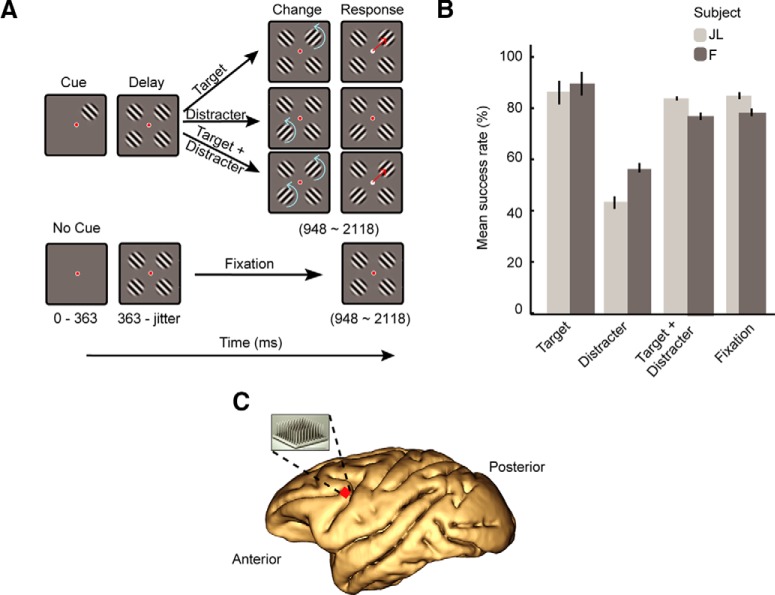
Task, methods, and behavior. ***A***, Experimental task design. Top, Attention trials. While the animals maintained fixation on a central point (red color represents gaze location), an initial target cue (Gabor grating at 100% contrast) was randomly presented in one of four quadrants on a computer screen. Three additional stimuli with identical contrast and orientation then appeared in the remaining quadrants of the screen (distracters). The animals needed to covertly attend to the cued stimulus to correctly saccade toward it after it rotated 90° (blue arrows). Bottom, Interleaved fixation trials in which four identical stimuli appeared without an initial cue; animals needed to hold fixation on the central fixation point until the end of the trial. ***B***, Performance of both monkeys in the experimental task (12 recording sessions in Monkey JL; 11 sessions in Monkey F). ***C***, Microelectrode array implantation site: Recordings were collected from area 8a of the left LPFC of each animal.

Two healthy adult male *Macaca fascicularis* (“JL,” 7.8 kg; “F,” 7.6 kg) were trained on an oculomotor task on a computer screen to measure the effects of attention and normalization in area 8a of the prefrontal cortex (Fig. 1*A*). Monkeys received an allotted amount of fluid (fruit juice) as a reward for successfully completing trials in the task. Water was restricted during experimental and training days, but it was lifted on non-testing days. Overall health, mental state, physical hygiene, and body weight were monitored daily. The animals were not sacrificed for this study. All experimental parameters were approved by the McGill Animal Care Committee, and complied with the rules of the Canadian Council of Animal Care.

### Surgical procedures

Animals underwent surgical operations under general anesthesia using isoflurane administered via endotracheal intubation. We first implanted titanium head posts to restrain head motion during experiments. We then chronically implanted one 10 × 10 Utah multielectrode array (Blackrock Microsystems) in the left dorsolateral prefrontal cortex of each monkey. Arrays were positioned on the cortex anterior to the arcuate sulcus and posterior to the caudal end of the principal sulcus, otherwise known as area 8A in the macaque monkey (Petrides, 2005). A Cerebus array connector (Blackrock Microsystems) was then fixed to each of the animals’ skulls with titanium screws. These screws protruded through the scalp to facilitate easier access during each recording session. Specific surgical procedures are described in a previous study by Leavitt et al. (2013). Some data from this study were previously used in another study by our group (Tremblay et al., 2015). However, in the current study, we include additional data not yet examined from the attention trials and fixation trials of our task (described below).

### Recordings

We recorded neural data using a Blackrock Microsystems Cerebus Neural Signal Processor and Cereport Adapter (Blackrock Microsystems). We recorded from one of three blocks of 32 channels on the 96-channel array; these blocks were fixed across all experimental sessions.

The broadband signal was bandpass filtered (0.3–7.5 kHz) and digitized to 16 bits at a sampling rate of 30 kHz. We detected individual spikes from this signal using a digital high-pass filter (250 Hz/four pole) in conjunction with a voltage threshold crossing of 4× root mean squared noise amplitude. These spikes and their associated waveforms were sorted offline manually using Offline Sorter v2.4 (Plexon Inc.). Single units (referred to as single neurons in this study) were distinguished from multi-unit activity, and were used for the remainder of the study. We isolated in 458 single units (199 across 11 sessions in Monkey F; 259 units across 12 sessions Monkey JL). We did not assume that recorded units were the same from day to day.

### Experimental setup

Gabor stimuli were projected onto a computer screen placed 1 m from the subjects’ eyes using a video projector (NEC WT610, 1024 × 768-pixel resolution, 85-Hz refresh rate). Experimental parameters of the task were controlled using custom-made software. Animal gaze positions were monitored using an infrared-based eye-tracking system (Eyelink 1000, SR Research). Saccade detections were accomplished by thresholding eye movement velocity at 25°/s. Subjects were seated in a standard primate chair, and were administered juice rewards via an electronic reward system (Crist Instruments) via a tube attached to the chair. A lever was installed to the chair and used by the monkeys to commence a trial. The chair + screen + pre-amplifier setup was shielded from electromagnetic interference with a Faraday cage.

### Experimental task

The paradigm consisted of two classes of experimental trials: attention trials, and fixation trials (Fig. 1*A*). In both trial types, subjects initiated a trial by fixating within 2° of a central fixation dot and depressing a lever, which had to be depressed for the entire length of a trial. Following a fixation period of 110 ms, the trial protocols diverged. The animals performed several hundred trials of randomly interleaved attention and fixation trials in each recording session. Successfully completed trials of either type yielded a juice reward and were followed by a 300-ms intertrial interval, while error trials had a 3000-ms waiting time with a black screen and no reward. The rule for all trial types can be succinctly stated as follows: saccade quickly to a cued change, otherwise maintain fixation.

### Attention trials

In attention trials, after the 110-ms fixation period, a circular Gabor stimulus (the “cue”), 1.5° wide with a spatial frequency of 2.5° and tilted at 45°, was presented at one of four possible positions around the fixation point, selected at random: an angle of 45° (top left), 135° (top right), 225° (bottom right), or 315° (bottom left), at 5° eccentricity. The cue indicated where on the screen they should covertly direct spatial attention while maintaining fixation in the center of the screen. After 363 ms of cue presentation (cue epoch), three distractor stimuli identical to the cue appeared at the remaining three positions, and all four stimuli (three distracters and cue) remained fixed on the screen for a random interval [585,1755 ms] (delay epoch), after which the trial would proceed in three possible ways, chosen randomly. (1) In “target” trials, the target orientation rotated 90° clockwise for 200 ms. Subjects had 400 ms following the onset of the target rotation to saccade to the target to successfully complete the trial. (2) In “distracter” trials, the 90° clockwise rotation occurred in the distractor stimulus diagonal to the target, and the monkey was required to maintain fixation to successfully complete the trial. (3) In “target + distracter” trials, changes occurred simultaneously in the target and distractor at the diagonal location. Subjects had to saccade to the target to successfully complete the trial, while an “error” trial was defined as the lack of a saccade to the target or the presence of a saccade to the distractor. Animals typically completed 200 of each type of attention trial in a single recording session.

### Fixation trials

On trial start in fixation trials, no cue stimulus was presented to the animals, and the screen remained blank for the duration of the cue epoch (363 ms) before four stimuli, identical to those used in attention trials, populated the four visual quadrants of the computer screen simultaneously. As in the attention trials, these four stimuli remained on the screen for a jittered duration of 585–1755 ms; however, in these trials, the subjects had no explicit instruction directing their covert spatial attention. A “correct” trial required the subject to maintain their gaze on the central fixation point for the duration of the trial.

### Calculation of chance performance levels

We calculated chance performance levels by considering the types of responses the animal can produce and the probability to get a reward if the animal chooses to randomly produce one of these responses after the change in the target. The target, distracter, and target + distracter trials comprise five options: saccade to one of the four targets, or withhold a saccade, yielding chance = 20%. Fixation trials comprise two options: saccade or withhold a saccade, yielding chance = 50%.

### Analyses

#### Single neuron selection

For the purposes of this study, we exclusively analyzed correct trials. For each neuron, we computed a non-parametric Kruskal–Wallis one-way ANOVA to compare the average firing rates of the units across all trials during the cue (for visual tuning) and delay (for attention tuning) epochs of the task. We computed firing rates in statically defined 300-ms windows in each trial. For cue epoch firing rates, we computed firing rates in a 300-ms window starting from 63 ms after cue onset. Delay epochs were jittered in length, ranging from 585 to 1755 ms. to include all available trials in our analysis, we computed firing rates using a window spanning 285–585 ms (i.e., the minimum jittered duration) after delay onset.

Of the 458 isolated single neurons, we included neurons that met the following criteria in our study: possessed an overall mean spike firing rate over 1 Hz; exhibited a significant change from pre-cue epoch baseline firing rate when presented with one of the four possible single stimuli (paired *t* test with threshold of α = 0.05, Bonferroni corrected), visually selective (Kruskal–Wallis test with threshold of α = 0.05), and attentionally selective (Kruskal–Wallis test with threshold of α = 0.05). A total of 236/458 (51.5%) units met each of these criteria. Spatial tuning for sensory and attention activity was assessed using mean firing rate responses during cue and delay epochs, respectively.

To determine sensory spatial tuning, we compared mean firing rates during trials in which the animals were presented a single stimulus in one of the four possible quadrants. If a stimulus in the hemifield ipsilateral to the recording site elicited a maximal response, that unit was deemed to be ipsilateral-tuned; similarly, a unit was classified as contralateral-tuned if a single stimulus displayed in the contralateral hemifield elicited a maximal response. For attention tuning, we compared mean responses during trials in which animals were attending to one of four simultaneously presented stimuli on the screen; the attended quadrant that elicited the maximum mean response in a unit was defined to be its preferred attended region of space. We excluded oculomotor-related neural activity during the saccade epoch of our task for this study.

#### Normalization analyses

To characterize the effects of sensory normalization in these isolated single units, we compared each unit’s firing rate during the cue epoch to its responses during the Fixation trials, in which four stimuli were presented simultaneously without a preceding cue. We computed firing rates during the cue epoch as explained above using windows spanning 63–363 ms after cue onset. We computed firing rates during fixation trials using identical parameters: 300-ms windows 63 ms after multiple-stimulus onset.

#### Multiple stimulus response index

We quantified the normalization response using a multiple stimulus response index (MSRI),

$$\mathrm{MSRI}=1-\frac{r_{\mathrm{all}}}{\sum_{i=1}^{4}r_{i}}$$

where *r_all_* is a unit’s mean response during Fixation trials, and *r_i_* is its mean response to one of the four possible presented single stimuli. Only units which exhibited a sublinear response to four simultaneously presented stimuli (i.e., MSRI > 0) were included for the remainder of the analysis (232/236 units; 98%).

#### Normalization response fitting

We compared unit firing rates to three simple models that each describe a candidate normalization scheme. Each model has zero free parameters. The first of these theoretical models was a linear summation (SUM):

$$\hat{r_{i}}=\sum_{j=1}^{4}r_{i,j}$$

Where *r_i,j_* is unit i’s mean response to one stimulus presented in one of four possible quadrants *j*, $\hat{r}$ is its mean response to four stimuli presented simultaneously, and *n* is the total number of recorded units. The second model described an averaging scheme (AVG):

$$\hat{r_{i}}=\frac{1}{4}\sum_{j=1}^{4}r_{i,j}$$

which is equivalent to the linear scheme, but with an additional 1/4 scalar multiplier included to average the sum of the four responses. Finally, we fit a WTA response model:

ri^=max(Ri);where⁢ Ri:{ri,1,ri,2,ri,3,ri,4}

We characterized the goodness of fits using root mean squared error (RMSE) for each of the three models.

**Figure 2.**
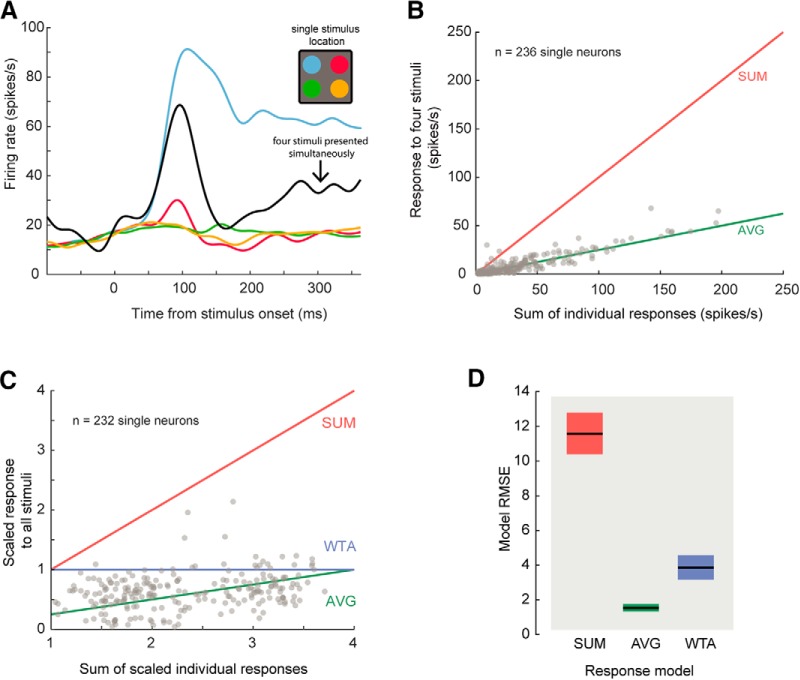
Normalization in area 8a. ***A***, Example single unit response to individually-presented stimuli (colored lines) and simultaneously-presented stimuli (black line). ***B***, Responses to multiple versus single stimuli. Each gray point is the sum of a unit’s spiking responses to four individually presented stimuli (*x*-axis) versus its firing rate when those four stimuli were presented simultaneously (*y*-axis). The red and green lines are predictions of linear additive (SUM) and averaging (AVG) responses. ***C***, Single unit spiking responses scaled to mean maximal response. Points are the same as in ***A***, but units with super-additive responses (those lying above the red line) were omitted (*n* = 4). Responses to all stimuli and responses to single stimuli were scaled (divided) by the maximum response to individual stimuli. WTA responses lie on the *y* = 1 line. ***D***, Bootstrap distributions of RMSEs of each of the three models. The AVG model yielded lowest RMSE (bootstrap *t* test between Averaging and WTA RMSE; samples; *p* < 1 × 10^−4^). Black lines are mean, and colored boxes are bootstrapped 95% CIs.

**Figure 3.**
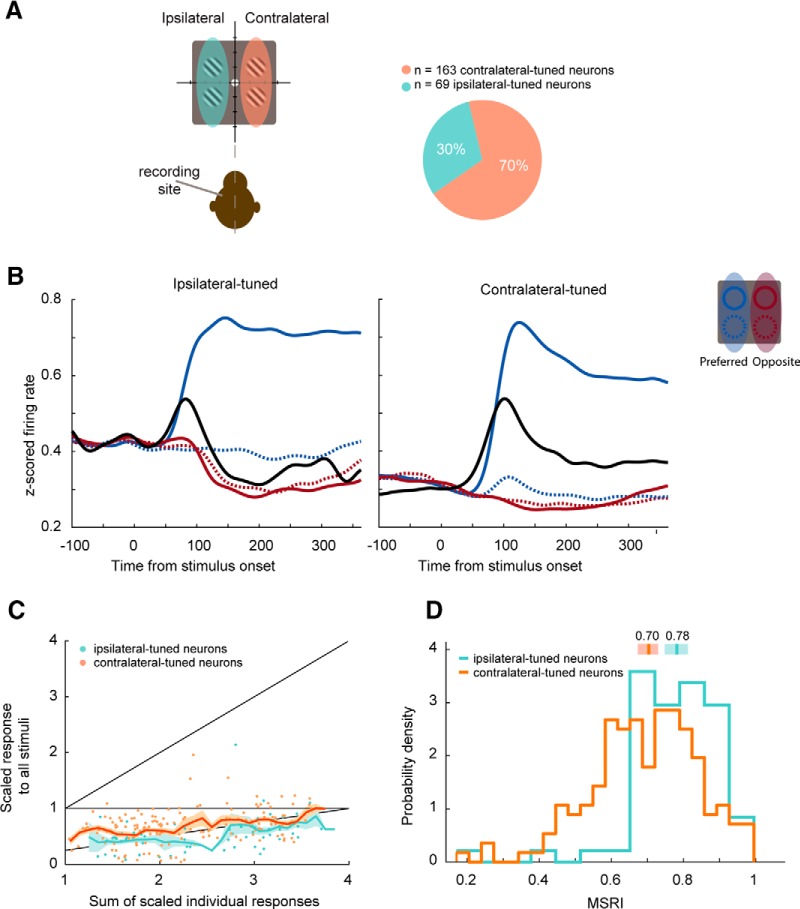
Tuned neural visual responses. ***A***, Individual unit spatial selectivity categorized by visual hemifield relative to recording site. ***B***, Average estimates of continuous firing rates (SDFs) for ipsilateral-tuned (left panel) and contralateral-tuned (right panel) populations. Colored lines are average responses to single stimuli presented in one of four possible quadrants; stimuli were shown inside a unit’s preferred quadrant (i.e., the stimulus which elicited a maximal response; solid blue), in a quadrant adjacent to the unit’s preferred quadrant within the same visual hemifield (dotted blue), adjacent quadrant in the opposite visual hemifield (solid red), or the quadrant located diagonal to the preferred quadrant (dotted red). Black lines are average population responses to these four stimuli when presented simultaneously. ***C***, Same as Figure 2*C*, but with units classified by their spatial tuning. Bootstrapped moving averages for each tuned population are displayed for visualization purposes (10,000 samples; window size 0.2; step size 0.05). Solid line denotes mean, shaded region denotes 1 SD of the bootstrap sample. ***D***, MSRI for ipsilateral-tuned and contralateral-tuned cells. Bars along top of plot denote median, shaded regions denote central 95% bootstrapped CIs (contralateral-tuned CI [0.67,0.73]; ipsilateral-tuned CI [0.75,0.81]).

To graphically visualize all three models simultaneously for Figures 2, 3, we scaled each *i*th unit’s $\hat{r}$ and *R* by $\max(R_{i})$. We then plotted each unit’s scaled $\hat{r}$ against the sum of its scaled *R*. Units which lie on the horizontal y=1 line exhibited a WTA response; units which lie on the y=x line exhibited a linear response; and units laying on the y=1/4x line exhibited an averaging response.

#### Attention analyses

We characterized attention versus no-attention normalization responses by comparing firing rates during the fixation trials (no cue, and therefore no directed attention), and delay epoch of attention trials (four possible quadrants to allocate spatial attention). We repeated the normalization analyses described above replacing the fixation trial firing rates ($\hat{r}$) with rates computed during the delay epoch of the task. The monkeys covertly attended to one of four possible cued quadrants during the delay epoch in which four stimuli were presented simultaneously. For our analyses, we examined the attention response differences for each unit during “attend in” and “attend out” conditions. Attend in trials were trials in which the monkey covertly attended toward the preferred spatial quadrant of a neuron (i.e., the quadrant in which a single stimulus elicited the maximum sensory response). With three possible remaining quadrants to direct attention toward, there were thus three possible attend out trial conditions for each unit. For our study, unless otherwise stated, we used attend out trials in which the monkey attended toward the quadrant adjacent to its preferred quadrant, across the vertical meridian (i.e., directly left or right of the preferred quadrant). However, our results were robust to our choice of attend out trial conditions.

#### Attention WTA dynamics

We quantified each unit’s attention response with respect to its response to its preferred stimulus (i.e., maximal single-stimulus response). We first computed spike density functions (SDFs) to estimate continuous time-varying firing rates by convolving spike rasters with a Gaussian kernel with SD 15 ms. Following this, we normalized each unit’s SDF to its maximum mean firing rate during the cue epoch in trials where a single stimulus was presented in its preferred quadrant. This scaled SDF allowed us to evaluate how attending into the preferred quadrant of a unit dynamically modulates its response onset toward a WTA computation.

#### Anatomic clustering of spatial selectivity

To determine whether spatial selectivity is anatomically clustered, we first determined the preferred hemifield of each multiunit cluster recorded on each array electrode. We restricted the analysis to a single recording session from each block of electrodes such that there was only one multiunit cluster per electrode. Next, we computed Moran’s *I* (Moran, 1950; Zuur et al., 2007; Leavitt et al., 2018) across the entire array. Moran’s *I* is a measure of spatial autocorrelation, the degree of clustering or similarity among objects in space, defined as:

$$I=\frac{N}{\sum_{i}\sum_{j}w_{i,j}}\frac{\sum_{i}\sum_{j}w_{i,j}(X_{i}-\overset{-}{X})(X_{j}-\overset{-}{X})}{\sum_{i}\left(X_{i}-\overset{-}{X}\right)}$$

where *N* is the number spatial units indexed by *i* and *j*; *X* is the variable of interest; *X̅* is the mean *X*; and *w_ij_* is an element of a matrix of spatial weights. Values of *I* range from −1 to 1. Positive values of Moran’s *I* indicate that similar feature values are spatially clustered, while negative values of Moran’s *I* indicate that similar feature values are spatially repellant or dispersed. Moran’s *I* was computed iteratively, extending the radius of included locations (the spatial radius) each time, until the whole array was included. This allowed us to determine the clustering of preferred location similarity across different spatial scales. For example, computing Moran’s *I* for the smallest cluster radius (400 μm) only included adjacent units, while computing it for the largest cluster radius included all units on the array. Significance was assessed using permutation tests.

#### State space neural trajectory analysis

In this study, we reduced the dimensionality of our 232-dimensional neuronal ensemble to two dimensions comprising average ipsilateral-tuned activity along one axis, and average contralateral-tuned activity along an orthogonal axis. Single-neuron SDFs were first scaled to their average maximal response, as described above. We then combined these single-neuron SDFs to compute grand mean responses to each condition (four attention conditions and one fixation condition) for ipsilateral-tuned and contralateral-tuned populations. These two grand mean population responses were plotted against each other for each condition, with time along a third orthogonal axis to display how these neural trajectories evolve during each trial condition.

#### Population spatial decoding

To assess visuospatial information content in the recorded population during the cue and delay epoch s of the task, we constructed a pseudo-population of single units from which to decode the location of the cue, and locus of covert spatial attention (quadrant of the screen). We analyzed successfully-completed attention trials of all types (target, distracter, target + distracter). Note that because the trial format for target, distracter, and target + distracter trials are identical until after the delay period (Fig. 1*A*), information about the type of attention trial is irrelevant to the decoding analyses. We sampled firing rates from 400 trials (100 trials × 4 quadrant trial conditions) for each unit. We extracted (decoded) information from this high-dimensional neural data using multinomial logistic regression with L2 regularization trained on these pseudo-populations (glmnet algorithm; Friedman et al., 2010). We repeated the below described decoding procedures, controlling for ensemble size used in the regression by subsampling the number of contralateral neurons to match the number of ipsilateral neurons (i.e., *n* = 69), and found quantitatively and qualitatively similar results. The results and figures in the main text show analyses using the entire recorded sample (69 ipsilateral and 163 contralateral neurons) without subsampling the contralateral population. Chance decoding was computed by training the model using shuffled trial condition labels, and testing on intact labels. We found that shuffled decoding accuracy closely matched theoretical chance decoding accuracy of 25% (i.e., guessing 1/4 quadrants correctly).

#### Sensory and attention information similarity

To measure the similarity between sensory and attention representation in these units, we trained a classifier on firing rates during the cue epoch, and tested on firing rates during the delay epoch. Firing rates were computed in 300-ms time windows (the same cue and delay epoch windows used in the single neuron analyses). We performed a nested k-fold cross-validation to optimize the regression weights. The dataset was first split into k = 5 partitions with 4/5 partitions used as the training set and the remaining 1/5 held out as the test set. Within this training set, we split the data again into k = 5 sub-partitions with 4/5 sub-partitions used to tune the regularization hyper-parameter by testing on the remaining 1/5 sub-partition. With the optimized model we then used the original test set to assess decoding performance. We then repeated this procedure 1000 times using newly sampled trials for each neuron on each iteration.

#### Temporal evolution of population responses

Using the classifiers trained on firing rates computed during the cue epoch described above, we then tested on sliding windows of sampled firing rates during the delay epoch to predict the location of covert spatial attention. Sliding windows had a boxcar shape (width 25 ms, step size 25 ms), and firing rates were computed in a trailing fashion, i.e., at time step t, the boxcar window integrated spiking information from *t*-25 ms to *t*. We chose bin widths of 25 ms after repeating the analyses for bin sizes of 5 ms through 35 ms, in increments of 5 ms. We found that decoding accuracies began to saturate at 100% at 25 ms, and thus used bin widths of this size for our temporal analyses.

#### Delay epoch decoding

As with previous decoding analyses, we used linear classifiers (glmnet multinomial logistic regression with L2 regularization) to decode the locus of spatial attention during the delay epoch when the cued stimulus and distracters were both present on the screen. The decoding accuracy of these classifiers could be used as a proxy for information content available to a downstream neuron to be read out (Moreno-Bote et al., 2014; Tremblay et al., 2015; Boulay et al., 2016). Three different configurations of neuronal ensembles were used to train the decoders. These were ensembles that comprised: exclusively ipsilateral-tuned neurons (69 neurons); exclusively contralateral-tuned neurons (163 neurons); or both ipsilateral-tuned and contralateral-tuned neurons (232 neurons). We found similar results when controlling for either RF size of each neuron included in the classifier, as well as number of neurons in each population. Firing rates were computed in bins of 25 ms, and stepped in time by 25 ms, as before.

#### Bootstrapping procedures

All nonparametric bootstrapping statistical tests were two-tailed. Unless otherwise stated, all bootstrap tests used 10,000 samples with replacement (1000 samples for decoding analyses). Two bootstrap-sampled distributions were deemed statistically different if 97.5% of their bootstrap distributions were non-overlapping (α = 0.05). When possible, *p* values for bootstrap statistical tests are reported exactly. However, when the observed *p* value is lower than the lowest possible *p* value achievable given our bootstrapping procedures (i.e., *p* = 10^−4^ for 10,000 samples or *p* = 10^−3^ for 1000 samples), then we report either *p* < 10^−4^ or *p* < 10^−3^, respectively (Table 1).

**Table 1.** Statistical table

	Data structure	Type of test	Power/CIs
a	Three non-negative continuous distributions of model fits' RMSE	Bootstrap *t* test	95% CIs; AVG: [1.29,1.80]; WTA: [3.05,4.69]; SUM: [10.14,13.00]
b	Three non-negative continuous distributions of model fits' RMSE	Bootstrap *t* test	95% CIs; AVG: [1.14,1.80]; WTA: [4.87,7.97]; SUM: [13.92,19.44]
c	Three non-negative continuous distributions of model fits' RMSE	Bootstrap *t* test	95% CIs AVG: [1.22,1.89]; WTA: [1.66,2.24]; SUM: [7.29,9.69]
d	Non-negative continuous within the range [0,1]	Non parametric Mann–Whitney *U* test	95% CIs; ipsilateral MSRI: [0.75,0.81]; contralateral MSRI: [0.67,0.73]
e	Integers within the range [1,4]	Bootstrap *t* test	95% quantile interval ipsilateral: [2.64,3.15]; contralateral: [2.24,2.58]
f	Spearman’s correlation coefficient within the range [–1,1]	Bootstrap *t* test	95% CI [0.06,0.29]
g	Proportions within the range [0,1]	χ^2^ test	*Post hoc* power: 35.4%
h	Spearman’s ρ within the range [–1,1]	Bootstrap *t* test	95% CI [0.41,0.61]
i	Spearman’s ρ coefficient within the range [–1,1]	Bootstrap *t* test	95% CI [–0.44,–0.22]
j	Two continuous non-normal distributions within the range [0,1]	Bootstrap *t* test	95% CI of MSRI; purely inhibitory: [0.783,0.840], mixture: [0.643,0.682]
k	Two continuous non-normal distributions within the range [0,1]	Bootstrap *t* test	95% CI of MSRI; purely inhibitory: [0.783,0.840], purely excitatory: [0.729,0.795]
l	Two continuous non-normal distributions within the range [0,1]	Bootstrap *t* test	95% CI of MSRI; mixture: [0.643,0.682], purely excitatory: [0.729,0.795]
m	Proportions within the range [0,1]	*Z* test for proportions	95% CI [71.39,80.20]
n	Proportions within the range [0,1]	*Z* test for proportions	95% CI [67.74,77.82]
o	Normal distribution	*t* test	95% CI [0.035,0.215]
p	Normal distribution	*t* test	95% CI [0.164,0.257]
q	Two continuous normally distributed distributions	Bootstrap *t* test	95% CIs; contralateral slope: [0.81,0.96]; ipsilateral slope [0.59,0.65]
r	Paired non-negative RMSE	Bootstrap *t* test	95% CIs; RMSE; AVG: [2.04,3.08]; WTA: [3.81,6.43]
s	Paired non-negative RMSE	Bootstrap *t* test	95% CIs; RMSE; AVG: [1.48,2.20]; WTA: [6.40,11.04]
t	Paired non-negative RMSE	Bootstrap *t* test	95% CIs; RMSE; AVG: [1.44,2.27]; WTA: [2.18,3.04]
u	Paired non-negative RMSE	Bootstrap *t* test	95% CIs; RMSE; AVG: [2.25,3.54]; WTA: [1.83,2.40]
v	Two continuous normally distributed distributions	Bootstrap *t* test	95% CIs; ipsilateral slope: [–2.588,–1.412]; contralateral slope: [–1.388,–0.212]
w	Two continuous normally distributed distributions	Bootstrap *t* test	95% CIs; ipsilateral slope: [0.104,0.496]; contralateral slope: [0.204,0.596]
x	Two continuous non-normal distributed distributions	Bootstrap *t* test	95% CIs; ipsilateral WTA index: [0.49,0.69]; contralateral WTA index [0.71,0.82]
y	Two continuous normally distributed distributions	Bootstrap *t* test	95% CIs; ipsilateral slope [–2.38,–0.42]; contralateral slope: [–2.784,–1.216]
z	One continuous normally distributed distribution	Bootstrap *t* test (test if different from zero)	95% CI [–0.18,0.08]
aa	One continuous normally distributed distribution	Bootstrap *t* test (test if different from zero)	95% CI [–0.10,0.13]
ab	Two continuous non-normal distributed distributions	Bootstrap *t* test	95% CIs; ipsilateral WTA index: [0.40,0.57]; contralateral WTA index [0.60,0.73]
ac	One continuous normally distributed distribution	Bootstrap *t* test (test if different from zero)	95% CI [0.010,0.066]
ad	Two continuous normally distributed distributions	Bootstrap *t* test	95% CIs; distance from cue activity shortly after distractor onset; ipsilateral: [0.19,0.20]; contralateral: [0.10,0.11]
ae	Two continuous normally distributed distributions	Bootstrap *t* test	95% CIs; distance from cue activity after sustaining attention; ipsilateral: [0.16,0.18]; contralateral: [0.09,0.11]
af	One continuous normally distributed distribution	Bootstrap *t* test (test if different from zero)	95% CI [0.061,0.149]
ag	Two non-negative distributions within the range [0–100]%	Bootstrap *t* test	95% CIs; ipsilateral trial accuracy: [–48,52]; contralateral trial accuracy: [78,82]

For information regarding the statistical procedures, please see the main text.

## Results

We trained two male macaque monkeys to direct gaze to (fixate) a dot at the center of a screen and covertly attend to a grating stimulus (the target) located in one of four possible screen quadrants while ignoring distracters located in the other quadrants. The location of the target was indicated by a cue appearing at the beginning of every trial. When the target changed orientation, the animal made a saccade toward it (attention trials; Fig. 1*A*, top). These attention trials were randomly interleaved with trials of a fixation condition wherein the four stimuli appeared simultaneously on the screen and the animal was required to maintain fixation until the end of the trial (Fig. 1*A*, bottom). Both subjects were trained to a level of task performance well above chance (20% for target, distracter, and target + distracter trials; 50% for fixation trials; see Materials and Methods; Fig. 1*B*). We implanted a multielectrode array (Utah array, Blackrock Microsystems) in the left LPFC of each monkey (Fig. 1*C*) and recorded the simultaneous activity of multi-units and single units (referred to here as single neurons) while the animals performed the tasks. We isolated 458 single neurons across 23 recording sessions (248 neurons across 11 sessions in monkey F, 210 neurons across 12 sessions in Monkey JL). Using firing rates computed in each trial during the cue epoch (300-ms time bin; 63–363 ms after cue onset) and sustained attention (i.e., delay) epoch (300-ms time bin; 285–585 ms after distracter onset), we found that 236/458 (51%) of these neurons were tuned for both the cue location (sensory tuning) and the allocation of attention (attentional tuning; assessed using Kruskal–Wallis ANOVA with a threshold of α = 0.05; see Materials and Methods). For the remainder of our analyses, we primarily focused on single neurons across all recording sessions that were selective during both the sensory (cue tuned) and delay (attention tuned) periods.

### Sensory normalization in LPFC area 8a

In a given attention trial condition, the cue was presented alone at the start of the trial, allowing us to examine the responses of the neurons to single stimuli presented in different quadrants of the visual field. The responses in the fixation trials allowed us to quantify the responses to all the stimuli presented together (Fig. 1*A*, bottom). Figure 2*A* shows an example neuron’s response to a lone cue stimulus presented in each of the four quadrants. The cue evoked the strongest response when presented in the upper left visual quadrant, and evoked minimal change from baseline when shown in the other quadrants. When all the stimuli were presented together the response was in between the maximal response to the cue on the upper left quadrant and the responses to the cue in the other quadrants. For each cell, we computed responses during the cue epoch or equivalent time window during fixation trials when the four stimuli were presented together. We tested whether the responses of single neurons to multiple stimuli equaled the sum responses to those stimuli presented alone, as predicted by a linear SUM model (Fig. 2*B*, SUM, red line). The vast majority of our tuned units’ (232/236; 98%) responses to multiple stimuli were sublinear and resembled those predicted by an average model computation (AVG, green line).

To further explore this issue, we transformed the data in Figure 2*B* by scaling each unit’s activity by its mean response to the cue that evoked the strongest response (preferred location or stimulus; Fig. 2*C*). This enabled the comparison of responses to a WTA response model prediction (blue line). WTA can be considered as a form of response normalization wherein a unit’s response to multiple stimuli is equal to its response to the preferred stimulus alone. We computed the RMSE from the data to the prediction of the SUM, AVG, and WTA models. Of these three response configurations, the AVG computation yielded the lowest RMSE (bootstrap *t* test, *p* < 10^4^,^a^ Bonferroni corrected; Fig. 2*D*). This demonstrates that neurons in area 8A undergo response normalization when multiple stimuli are presented in the visual field and that, from the three models considered, the AVG model best describes the computation.

### Spatial tuning and normalization

We divided our sample into two subsets: neurons that produced a maximal response when the single stimuli were presented ipsilateral to the recording hemisphere and neurons that produced a maximal response when the stimulus was presented contralateral to the recording hemisphere (referred to as ipsilateral and contralateral neurons, respectively; Fig. 3*A*,*B*). Figure 3*C* shows the same plot as Figure 2*B* with each neuron labeled according to its visuospatial tuning. By comparing RMSE between models, we found that both populations of tuned neurons were best described by an AVG response (bootstrap *t* test on RMSE, *p* < 10^−4^,^b,c^ Bonferroni corrected). We further quantified the strength of normalization in each neuron using a MSRI,

$$\mathrm{MSRI}=1-\frac{r_{\mathrm{all}}}{\sum_{i=1}^{4}r_{i}}$$

where *r_all_* is a unit’s average response to the four stimuli presented simultaneously, and $r_{i}$ is its response to a single cue stimulus presented in quadrant *i* on the screen (*i* = 1 … 4 for the four quadrants). If a unit’s response to all stimuli (*r_all_*) equals the sum of the responses to each stimulus alone ($\sum r_{i}$) then MSRI is 0; if *r_all_* is greater than $\sum r_{i}$, MSRI is <0; and if *r_all_* is lower than $\sum r_{i}$, MSRI is between 0 and 1. For our task with four stimuli, an average (AVG) response would occur when *r_all_* =$\frac{1}{4}\sum r_{i}$, and MSRI = 0.75. Although the distributions of MSRI overlap, the average MSRI was greater for ipsilateral neurons (median = 0.78, 95% confidence interval (CI) [0.75,0.81]) than for contralateral neurons (median = 0.70, 95% CI [0.67,0.73]; Mann–Whitney *U* test, *z* = 4.32, *p* = 7.97 × 10^−6^;^d^ Fig. 3*D*). This indicates a stronger response normalization in ipsilateral than in contralateral units.

### Receptive field size and normalization

**Figure 4.**
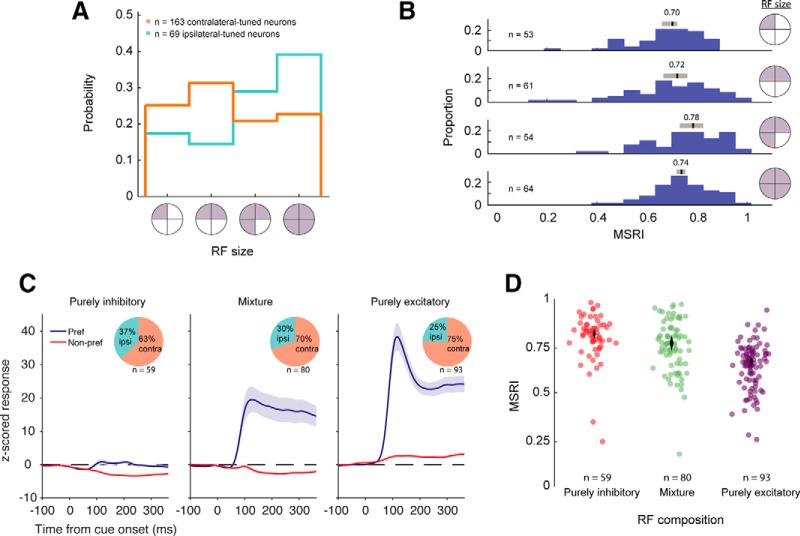
Receptive field properties. ***A***, Distributions of receptive field sizes (number of quadrants) for ipsilateral and contralateral neurons. A quadrant of the visual field was classified as being part of a unit’s receptive field if a singly presented stimulus in that quadrant elicited a response (excitatory or inhibitory) different from pre-stimulus baseline. ***B***, Corresponding MSRI of units with a given receptive field size. Medians (black vertical lines) were computed using 10,000 bootstrap samples, and gray bars indicate the central 95% CIs of the distribution of medians. CIs for top row: [0.65,0.72], second row: [0.66,0.76], third row: [0.73,0.81], and fourth row: [0.71,0.76]. ***C***, Receptive field configurations. Singly presented stimuli may either excite or suppress neuronal activity relative to baseline. Thus, the receptive field of a given neuron can be (1) purely inhibitory, (2) purely excitatory, or (3) a mixture of both. Neuronal responses (z-scored to baseline) were combined for each of the three possible groups. Preferred responses were the responses to stimuli which elicited the greatest response (or the stimuli which elicited the least amount of suppression, in the case of the purely inhibitory RFs). Non-preferred responses are the average response to the three stimuli, excluding the preferred stimulus. Insets show proportion of ipsilateral-tuned and contralateral-tuned cells in each group. ***D***, MSRI of units with RF compositions shown in ***C***. MSRI was greater in units with a greater proportion of inhibitory receptive quadrants. Each dot is one single unit. Dots are horizontally jittered with reduced opacity for clarity. Vertical length of diamonds are 2.5th and 97.5th percentile CIs of the bootstrapped distributions (10,000 samples) of medians. CIs for purely inhibited cells: [0.79,0.83], cells with a mixture of excited and inhibited activity: [0.73,0.79], and purely excited cells: [0.64,0.68].

The difference in normalization between contralateral and ipsilateral neurons could be explained by differing RF properties between units, such as RF size. Here, we consider the RF as the region of the visual field that is modulated by the appearance of the single cue and includes both excitatory (RFe) and inhibitory (RFi) regions. To investigate this issue, we first estimated the size of each unit’s RF (pooling across both RFe and RFi) by examining whether individually presented stimuli in each quadrant modulated the firing rate relative to the pre-stimulus baseline response (paired *t* test with threshold of α = 0.05, Bonferroni corrected; Fig. 4*A*; see Materials and Methods). On average, ipsilateral-tuned units had RFs spanning more quadrants (bootstrapped mean ± SD: 2.9 ± 0.1 quadrants) than contralateral-tuned units (2.24 ± 0.09 quadrants, bootstrap *t* test, *p* < 10^−4^).^e^ However, the overall size of a unit’s RF was only weakly correlated with its MSRI (Spearman’s ρ = 0.18, bootstrap 95% CI [0.06,0.29], *p* < 10^−4^;^f^ Fig. 4*B*), and thus it is unlikely to fully account for the difference in MSRI between the two populations.

It is possible that the difference in normalization between the populations are related to the specific composition of the RF in terms of excitatory and inhibitory regions. Specifically, the suppressed response to many stimuli may depend on whether specific regions of the RF are excitatory (RFe) or inhibitory (RFi). We determined whether a region was excitatory or inhibitory by quantifying the difference in responses between a single cue, and pre-cue baseline when the animal was only fixating the center point with no stimulus present. Indeed, in our recorded population we obtain a heterogeneous sample of RF compositions: purely inhibited (all responses below baseline), purely excited (all responses above baseline) and mixture of the two (Fig. 4*C*). However, we found a trend for a larger proportion of ipsilateral tuned neurons to be purely inhibited, and an opposite trend of a higher proportion of contralateral neurons to be purely excited that did not reach statistical significance (χ^2^ test *p* = 0.09^g^).

Whereas the size of the RFi was positively correlated with the MSRI (Spearman’s ρ = 0.50 ± 0.05, bootstrap mean ± SD, *p* < 10^−4^),^h^ the size of a unit’s RFe was negatively correlated with the MSRI (Spearman’s ρ = –0.34 ± 0.06, bootstrap mean ± SD, *p* < 10^−4^).^i^ Thus, the larger a neuron’s RFi (measured as number of quadrants), the more normalization it exhibited. Conversely, a neuron with a larger RFe was likely to show weaker normalization. In agreement with this result, the median MSRI for neurons with purely RFi was greater than for neurons with purely RFe (bootstrap *t* tests between MSRI distributions, purely inhibitory vs purely excitatory: *p* < 10^−4^,^j^ purely inhibited vs mixture: *p* = 0.012,^k^ mixture vs purely excitatory: *p* < 10^−4^;^l^ Fig. 4*D*). This result suggests that the extension of the RFe and RFi, determined using singly presented stimuli, relates to the amount of response normalization a given neuron undergoes when multiple stimuli are presented. Furthermore, contralateral neurons, with larger RFe regions and smaller RFi regions, undergo less normalization than their ipsilateral counterparts.

**Figure 5.**
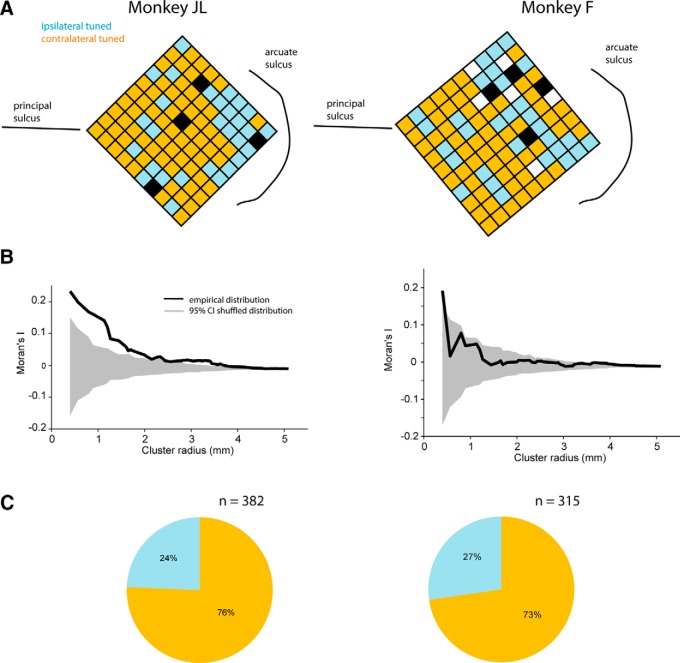
Anatomic clustering of spatial tuning. Multielectrode array data from Monkey JL (left column) and Monkey F (right column). ***A***, Multi-unit spatial tuning (ipsilateral or contralateral) on each electrode of the array when using three recording sessions (one per block of 32 electrodes; see Materials and Methods) from each monkey. Black squares are inactive channels, and white squares are channels in which no tuning was present (for details, see Materials and Methods). ***B***, Spatial autocorrelation of tuned clusters on recording array using Moran’s *I*. Black curves are empirical distributions and gray shaded regions are shuffled 95% null distributions. Although both subjects showed significant clustering at the smallest cluster size, Monkey JL exhibited significant clustering at larger spatial scales than Monkey F. ***C***, Distribution of multi-unit spatial tuning across all recording sessions (12 sessions in Monkey JL, and 11 sessions in Monkey F).

Another possibility that may account for the differences in response normalization is that the populations of ipsilateral and contralateral neurons mutually inhibit each other, with the strength of the inhibition being proportional to the size of the neuronal pool. Previous studies have shown that contralateral neurons are more numerous than ipsilateral neurons in this area (Lennert and Martinez-Trujillo, 2013; Bullock et al., 2017). We observe this again in the current study, using the multiunit activity obtained from each electrode to determine its tuning (contralateral or ipsilateral). Figure 5*A* shows the locations of ipsilateral (cyan) and contralateral (orange) units on the microelectrode array for each animal; a significantly larger proportion of neurons are contralateral compared to ipsilateral (*Z* test for proportions, H_0_: 50% ipsilateral tuned, 50% contralateral tuned; Monkey JL, *Z* = 10.2, *p* < 10^−4^;^m^ Monkey F, *Z* = 8.2, *p* < 10^−4^;^n^ Fig. 5*C*) . We also computed Moran’s *I*, an index of spatial autocorrelation, to quantify clustering of these two groups of neurons on the cortical surface, and observed significant clustering in both animals; neurons of a given type (e.g., contralateral) are more likely to be surrounded by neurons of the same type than by neurons of the opposite type (Fig. 5*B*, Moran’s *I*, black line above the gray null distribution). This trend was more evident in animal JL for different cluster radiuses, while in animal F the trend only occurred for small cluster radiuses.

### Effects of attention on normalization responses

**Figure 6.**
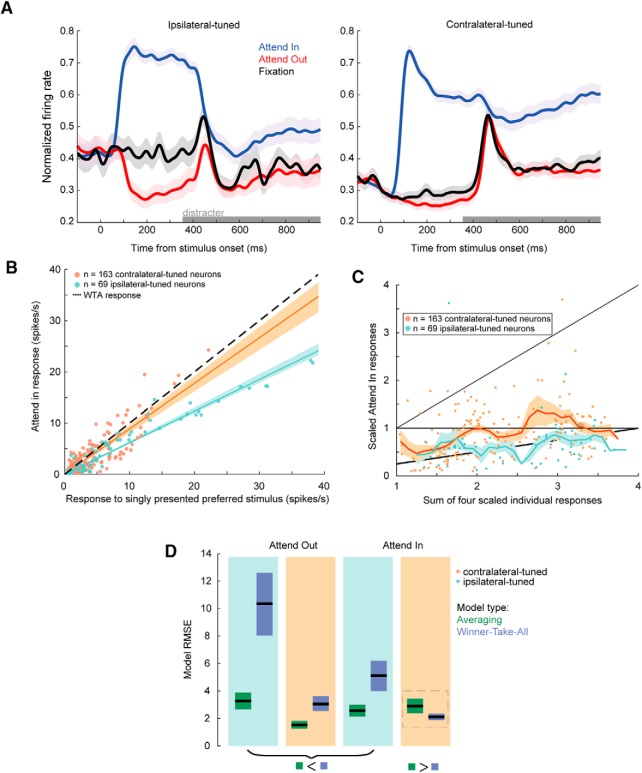
Ipsilateral and contralateral population attention responses. ***A***, Bootstrapped population average SDFs for ipsilateral (left panel) and contralateral populations (right panel). Attend in, attend out, and fixation trial responses are shown in blue, red. and black, respectively. Attend out trial conditions were averaged across the three non-preferred locations. Single neuron spike trains were trial-averaged, convolved with a Gaussian kernel (15-ms SD), z-scored, and finally averaged within each ipsi/contra population. ***B***, Comparison between each unit’s response to a single stimulus presented in its RF center during the cue epoch (*x*-axis) vs its attend in response during the delay epoch when distracters were present (*y*-axis). Dotted line is when a unit’s attend in response matches its response when presented that stimulus alone (i.e., WTA response). ***C***, The sum of each unit’s responses to individual stimuli (*x*-axis) versus attend in responses (*y*-axis); *x*-coordinates of each point are identical to those in Figure 2*B*. ***D***, RMSE for WTA and AVG models during attend in and attend out conditions for ipsilateral-tuned and contralateral-tuned units. For attend out conditions, we used trials where the animals were attending to the quadrants adjacent to a given unit’s preferred quadrant and located in the opposite hemifield. We found similar results using attend out responses for the remaining two quadrants (i.e., either attending to the quadrant diagonal to the preferred quadrant, or the quadrant adjacent to the preferred quadrant and in the same hemifield) as well. RMSE for SUM models were omitted for clarity due to being much greater in magnitude compared to AVG and WTA model RMSE.

Stimuli presented during attention trials were identical to those presented during fixation trials. However, after the cue presentation during attention trials, when the four stimuli appear on the screen, the subjects covertly attended toward the cued stimulus in one quadrant while ignoring the three distracters located in the other quadrants (Fig. 1*A*, top). We characterized neuronal responses when attention was allocated to (1) the stimulus that evoked a stronger response when presented alone (preferred stimulus, attend in), (2) one of the non-preferred stimuli (attend out), and (3) the fixation point, when none of the four stimuli were cued (“fixation”). Ipsilateral and contralateral neuronal responses in these three different attention conditions are shown in Figure 6*A*.

Firing rates were on average higher during attend in trials than during attend out trials (Fig. 6*A*, blue and red lines, respectively). This effect was highly significant for both the ipsilaterally-tuned population (*t* test, *t*_(68)_ = 5.5, *p* = 6.2 × 10^−7^)^o^ and contralaterally-tuned population (*t* test, *t*_(162)_ = 7.15, *p* = 2.8 × 10^−11^).^p^ We found that attend in responses of contralateral neurons, but not ipsilateral neurons, were better described by a WTA model (a linear regression slope of 1 signifies a WTA response; Fig. 6*B*). The regression slope for contralateral neurons (median slope = 0.89; 95% CI [0.81,0.96]) was greater than that for ipsilateral neurons (median slope = 0.62; 95% CI [0.59,0.65]; bootstrap *t* test, *p* < 10^−4^).^q^ Figure 6*C* shows each unit’s sum of responses to individually presented stimuli versus its response to four stimuli during attend in trials. We computed the RMSE corresponding to each model prediction for contralateral and ipsilateral neurons during both attend in and attend out conditions. Attend in and attend out responses of ipsilateral neurons remained best-described by an AVG model (bootstrap *t* test on RMSE, attend in *p* < 10^−4^;^r^ attend out *p* < 10^−4^;^s^ Fig. 6*D*). For contralateral units attend out responses were also better described by an AVG model (bootstrap *t* test on RMSE, AVG vs WTA *p* = 3 × 10^−4^).^t^ However, contralateral attend in responses were best described by a WTA computation (dashed gray box; bootstrap *t* test, *p* = 0.01^u^). Thus, when the animals attended to the preferred stimulus, normalization in contralateral units shifted from AVG to WTA.

### Dynamics of sustained attention

**Figure 7.**
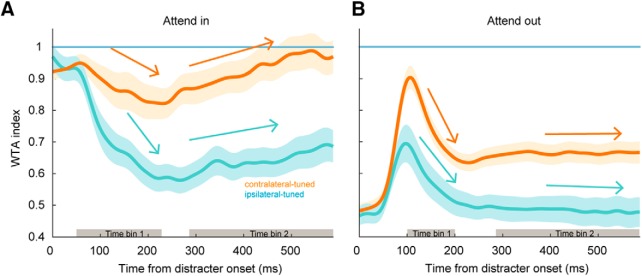
Single neuron characterization of attention dynamic responses. Bootstrapped average population WTA index (*y*-axis) over time (*x*-axis) for (***A***) attend in and (***B***) attend out conditions. We computed linear regression slopes (arrows) for the decaying, and sustained attention portions of the curves during time bins denoted by gray bars on the *x*-axis. Shaded error bars are 1 SD. Each line was computed using 10,000 bootstrap samples.

The previous analyses using firing rates averaged over statically defined time periods of the task overlooks the temporal dynamics of attentional modulation. We investigated this issue by first examining how responses during the attentional period change over time relative to the cue period responses. We scaled each unit’s SDF (Fig. 6*A*) by the mean response to its preferred stimulus (i.e., maximal response) to yield a time-evolving index. We called this index the WTA index to refer to changes in response during the attentional period relative to the response when the cue is presented alone (Fig. 7). A decrease in WTA index away from 1 indicates a stronger normalization response toward the AVG regime caused by distracters.

Both population WTA indices decreased following distracter onset in the attend in condition (Fig. 7*A*), indicating that responses to all stimuli decreased relative to the single target response. In other words, responses were normalized toward the AVG response on distracter onset. We computed the temporal rate of normalization in the ipsilateral and contralateral populations after distracter onset by fitting straight lines to the time evolving WTA indices (linear regression slopes computed during time bin 1, indicated by the left shaded gray bar along the *x*-axis; Fig. 7*A*). The ipsilateral population slope (cyan arrow; time bin 1) was more negative than the contralateral population slope (orange arrow; time bin 1; mean ± SD, ipsilateral: –2.0 ± 0.3 s^−1^; contralateral: –0.8 ± 0.3 s^−1^; *p* = 8 × 10^−4^,^v^ bootstrap *t* test). This shows that during attend in trials, ipsilateral neurons were more strongly normalized by the appearance of the distracters than contralateral neurons. Approximately 250 ms following this initial response decrease, there was an upward trend (time bin 2, indicated by the right shaded gray bar along the *x*-axis) toward a WTA response (dashed line), with positive average slopes for each population, not statistically different from one another (orange and cyan arrows aligned with bin 2; ipsilateral-tuned slope: 0.3 ± 0.1 s^−1^; contralateral slope: 0.4 ± 0.1 s^−1^; *p* = 0.18^w^, bootstrap *t* test). This suggests that the rate of response change toward the WTA regime when the animals maintained attention on the target is similar in both populations. However, the degree of suppression (normalization) caused by distracter onset is stronger in ipsilateral neurons.

During attend out trials, neural activity after distracter onset initially moves toward a WTA regime for both populations, however this increase was greater in magnitude for contralateral neurons (comparing means during time bin 1; bootstrap *t* test, *p* < 1 × 10^−4^;^x^ Fig. 7*B*). During attend out trials, since the initial cue was outside the preferred region of each neuron’s RF, the change in normalization during the delay epoch was likely due to a distracter populating the neurons’ RFs. After this initial increase, the WTA index promptly decreased in both populations. The modulation rate (linear regression slope during time bin 1) for both contralateral and ipsilateral units were similar and not significantly different from one another (ipsilateral: –1.4 ± 0.5 s^−1^; contralateral: –2.0 ± 0.4 s^−1^; *p* = 0.12^y^, bootstrap *t* test). After this initial rapid decay, normalization stabilized (Fig. 7*B*, time bin 2), and we found that linear regression slopes were not different from zero for either the ipsilateral (*p* = 0.42^z^, bootstrap *t* test) or contralateral population (*p* = 0.86^aa^, bootstrap *t* test) during this period. However, the normalization level at which ipsilateral responses stabilized was significantly lower than the level at which contralateral responses stabilized (both relative to the WTA line; *p* < 1 × 10^−4^,^ab^ bootstrap *t* test comparing means during time bin 2). This is likely a direct consequence of the initial response increase being stronger in contralateral relative to ipsilateral units and the decrease being of similar magnitude in both subpopulations.

**Figure 8.**
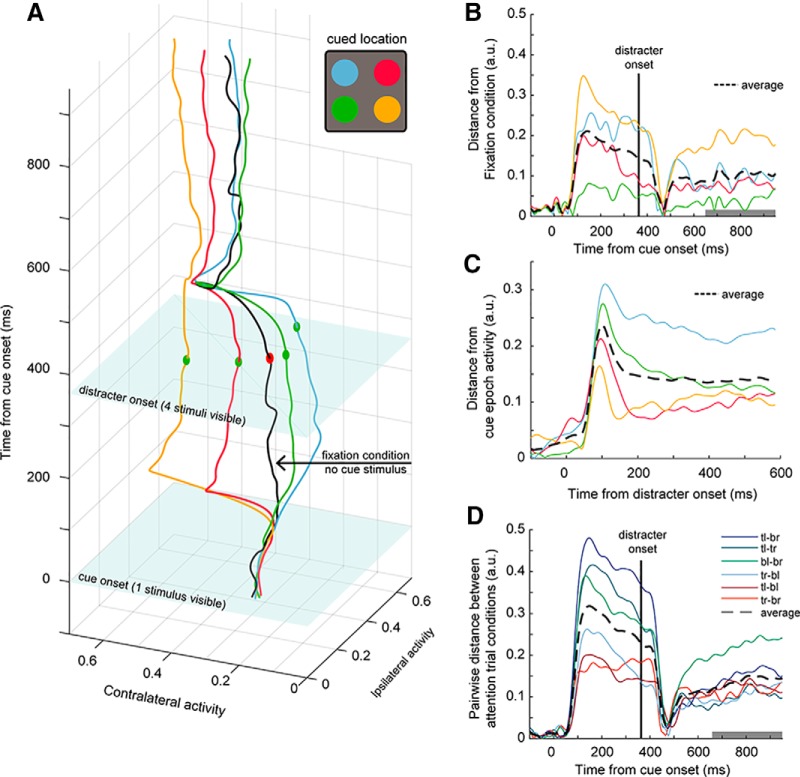
Temporal dynamics of attention. ***A***, State space trajectories. Average activity across contralateral neurons (*x*-axis) is plotted against average activity across ipsilateral neurons (*y*-axis) for each point in time (*z*-axis) and averaged within each trial condition. Colored lines are average activity during the four possible attention trial conditions, and the black line is the average activity during the Fixation trial condition in which no cue was presented. ***B***, Euclidean distance through time of each average attention trial condition trajectory (colored) from the fixation trajectory in ***A***. Dashed black line is the average of the four conditions. A linear regression slope was computed for the dashed line during the latter portion of the delay epoch (gray time bin). ***C***, Time-evolving Euclidean distance of each attention trial condition’s delay epoch activity (i.e., activity after distracter onset) from its respective mean activity during the cue epoch. Dashed line is the average of the four conditions. ***D***, Pairwise Euclidean distance between each attention trial trajectory in ***A***. Dashed line is the average of all the pairwise comparisons. Comparisons were made between conditions where the animal attended to the top left (tl), top right (tr), bottom left (bl), or bottom right (br) quadrants of the screen. A linear regression slope was computed during the latter portion of the delay epoch (gray time bin).

To make the temporal dynamics of the responses more intuitive, we applied a state space analysis (Murray et al., 2017; Ebitz et al., 2018) to the population of ipsilateral and contralateral neurons. We averaged the SDFs of neurons (scaled to the mean response to their preferred stimuli) in each subpopulation for the four attentional conditions. This effectively reduced the dimensionality of the dataset from *n* = 232 to 2 dimensions, with each dimension representing the activity of cells with opposing spatial tuning (contralateral or ipsilateral). The two horizontal axes in Figure 8*A* represent these two dimensions, the vertical axis represents time, and trials of different attentional conditions are color coded.

In this plot, by folding the trajectories around the “distracter onset” plane (corresponding to the time at which distracters appeared during the attentional conditions), one can compare how much the trajectories during the cue epoch and during the attentional delay epoch resemble each other. Identical cue and attentional delay trajectories for a given cue would indicate that attention operates to bring the population state toward an ideal WTA regime that fully filters out the neural activity evoked by distracters. On the other hand, if the trajectories in the attention conditions approximate the trajectory for the attention-free fixation condition, the network would be in a full normalization regime and attention would have no effect.

Note that during the cue period, no stimulus was displayed in fixation trials, and the animals were simply holding their gaze on the fixation point. Distances from the different cue/attentional conditions to the fixation condition depart from zero after cue onset and reached their maximum during the first 200–300 ms of the cue period (Fig. 8*B*). On distracter onset, neural trajectories converged toward the fixation condition trajectory at ∼470–480 ms. However, as attention was sustained on the target, trajectories diverge from fixation and the average separation between the attention conditions and fixation trajectories increased again over time. We computed a linear regression slope of the average distance as a function of time over the delay epoch (dotted black line, slope computed over gray shaded region) and found it to be significantly positive (bootstrap *t* test, 1000 samples, *p* < 0.001^ac^). This result indicates that population activity increasingly diverges from the fixation condition as attention is sustained on the target.

To determine how much the effect of attention migrates toward an ideal WTA regime, we compared each neural trajectory during the delay epoch (after distracter onset) to its respective mean trajectory during the cue epoch (Fig. 8*C*). This shows how population activity when attending toward a stimulus surrounded by distracters compares to its activity when the stimulus was presented in isolation (i.e., how closely does delay activity resemble a perfect WTA). Distracter onset (time = 0; Fig. 8*C*) caused neural trajectories to diverge quickly away from the WTA regime, with average ipsilateral trial trajectories (mean of blue and green lines 50–250 ms after distractor onset) displaced farther than average contralateral trajectories (mean of red and yellow lines 50–250 ms after distractor onset; bootstrap *t* test, *p* < 0.001^ad^). Following distracter onset, trial trajectories on average evolved toward, but did not fully reach a WTA regime (Fig. 8*C*, black dashed line). Trajectories of trial conditions in which attention was allocated toward the contralateral hemifield (i.e., average of red and yellow lines 285–585 ms after distractor onset) advanced closer to a pure WTA regime than trajectories of conditions in which attention was allocated to the ipsilateral hemifield (average of blue and green lines 285–585 ms after distractor onset; bootstrap *t* test, 1000 samples, *p* < 0.001^ae^). Importantly, neither of the trajectories fully reached a WTA regime but seemed to asymptote and stabilize during the sustained attention period.

To determine how neural trajectories corresponding to a given trial condition differ from other conditions over time, we also measured the pairwise Euclidean distances between each trial condition (Fig. 8*D*). We found that trajectories were highly similar during the baseline period, and maximally differ after cue presentation during the cue period. Following distracter onset, the trajectories converged to a similar state, and diverged again during the delay epoch when attention was sustained (i.e., the average separation between each trial condition’s neural trajectory increased again after they collapsed). Specifically, the linear regression slope of the average pairwise distance as a function of time during sustained attention was significantly positive (dotted black line, slope computed over gray shaded region; bootstrap *t* test, 1000 samples, *p* < 0.001^af^). This shows that as attention is sustained, neural trajectories become more divergent from each other.

### Population coding of sustained attention

Given the non-trivial translation from single neuron coding to population coding, it is unclear whether the effects we reported in averaged populations extend to the full neuronal ensemble activity. Furthermore, given the observed temporal modulation of firing rates and normalization, it is unclear whether the population code during the cue period generalizes to the code during the attention period. Linear classifiers have proven effective for applications such as comparing the information content in neuronal ensembles during different trial periods and determining the similarity between different coding regimes (Moreno-Bote et al., 2014; Leavitt et al., 2017). Thus, we used linear classifiers to assess how similar the population code is when the subject is attending to a target in the presence of distracters compared to when the target is presented alone. We can consider the activity profile and the code used during the cue period as corresponding to a perfect WTA regime. The more the code generalizes between the attentional and cue periods, the more similar sustained attention is to an ideal WTA computation, and consequently the distracters are filtered.

**Figure 9.**
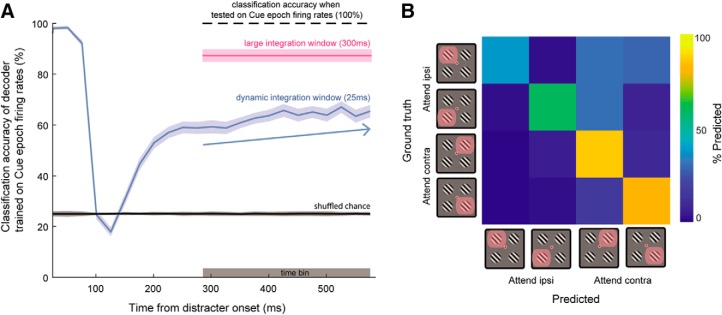
WTA decoding with sustained attention. ***A***, Linear classifier using a pseudopopulation of 232 single unit firing rates trained on the latter 300 ms of the cue epoch, then tested on: (1) firing rates computed in a 300-ms time window (pink shaded error bar, using firing rates integrated over gray time bin shown along *x*-axis), and (2) dynamic, trailing moving windows during the delay epoch (window = boxcar with width 25 ms; step size = 25 ms). Solid lines are mean classification accuracy, and shaded error is 1 SD of entire bootstrapped sample. Classification accuracy slowly increased after recovery from transient activity after distracter onset (linear regression computed on dynamic classification accuracy during time bin denoted by gray shaded region) with a slope of 0.02 ± 0.01 ms^−1^ (mean ± SD; blue arrow). ***B***, Example confusion matrix derived from the final time point of the blue curve in ***A***. Trials in which animals attended toward the ipsilateral hemifield were misclassified more than trials where they were to attend toward the contralateral hemifield (50 ± 3% vs 80 ± 3% correct).

We used a linear classifier to decode the locus of spatial attention; we trained our model on average firing rates integrated over a 300-ms window during the cue epoch (same window as in previous analyses; see Materials and Methods), and tested on average firing rates during a 300-ms window of the attention (delay) epoch. Using all 232 single neurons, the classifier achieved decoding accuracy of 87 ± 2% (Fig. 9*A*, pink line, test epoch firing rates computed in gray time bin). This shows that the population response with attention toward a target among distracters is similar to that of when the object was presented alone, suggesting an approximate WTA regime at the population level. We quantified the dynamics of this state similarity using the same classifier trained on the firing rates during the 300-ms cue epoch time bin and evaluated the time-evolving state similarity by testing the classifier on sliding windows of firing rates during the delay epoch (Fig. 9*A*, blue curve). We used a 25-ms boxcar window stepped by 25 ms to compute firing rates from the spike rasters. Decoding accuracy was nearly 100% immediately following distracter onset, likely because the neuronal population still contained residual cue-evoked activity and the distracter information had not yet reached PFC. At 80–90 ms after distracter onset, accuracy drastically fell toward chance level (∼25%; gray line is an estimate of chance level accuracy using shuffled permutation test). However, the decoding accuracy recovered and increased toward ∼60% shortly thereafter, and continued to steadily increase with sustained attention (linear regression slope of 0.02 ± 0.01% ms^−1^; mean ± SD; Fig. 9*B*, blue arrow, regression computed using gray shaded region). Therefore, with sustained attention the pattern of neuronal ensemble coding of the attended stimulus evolved over time, and resembles the WTA observed when the cue was presented alone.

We show a representative confusion matrix derived from test-set predictions made by a decoder at the final time bin of the sliding decoding window Figure 9*B*. Interestingly, the classifiers made a greater number of errors for trials in which attention was allocated toward the ipsilateral field than when attention was directed toward the contralateral field (50 ± 1% vs 80 ± 1%; bootstrap *t* test *p* < 10^−3^).^ag^ Importantly, our findings from Figure 9*A*,*B* were robust to controlling for the uneven population sizes of ipsilateral and contralateral neurons (data not shown); for this reason, we opt for including all recorded ipsilateral and contralateral neurons in this analysis as we believe this to be more representative of the underlying LPFC neuronal population. Thus, when attending toward a stimulus in the presence of distracters, the population read-out of information relevant to the contralateral hemifield is more effective than that of the ipsilateral hemifield. This is likely a resulting effect of the varying differences in magnitude of attentional modulation between the ipsilateral-tuned and contralateral-tuned populations (Fig. 6). Here, one may consider that if the same result would be obtained when recording from the same area in the opposite hemisphere, a downstream reader that read out information from both hemispheres would make few errors in this task.

### Decoding the allocation of attention

**Figure 10.**
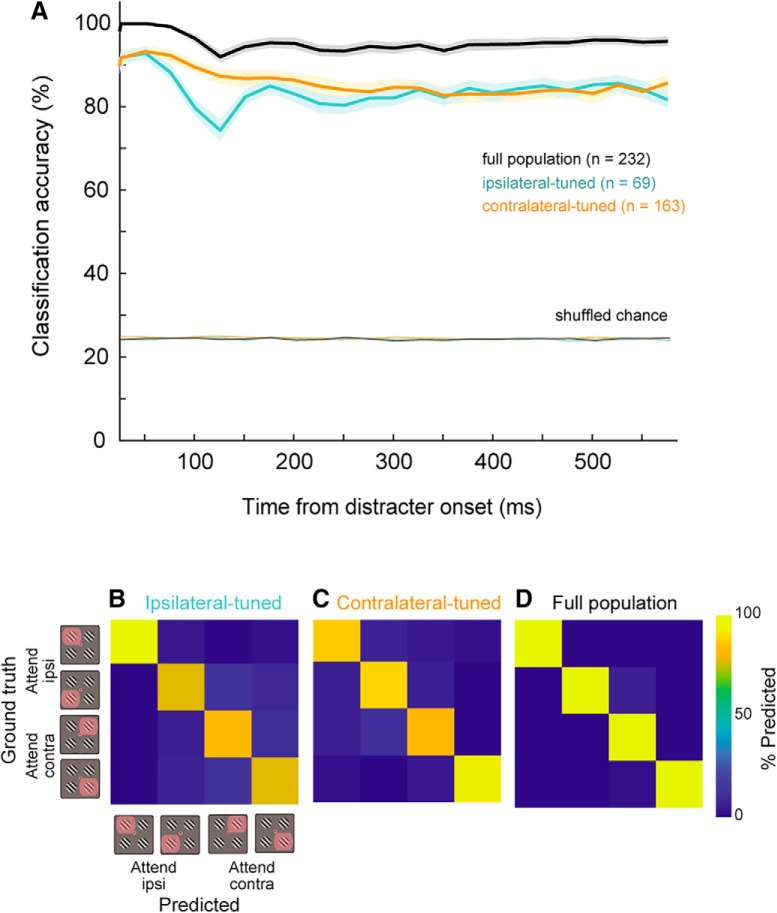
Ensemble decoding of locus of covert attention during delay epoch. ***A***, Linear classifier trained and tested on 25-ms trailing windows stepped by 25 ms during the delay epoch of the task. Bootstrapped average classification accuracies using ensembles comprising exclusively the ipsilateral-tuned population (blue) or contralateral-tuned population (orange), or an ensemble comprising both populations (black). Shaded error bars are 1 SD of the entire bootstrap sample. ***B–D***, Confusion matrices for the ipsilateral-tuned, contralateral-tuned and full population classifiers derived from the final time points of the curves in ***A***.

One may ask what the accuracy of the classifier would be if it were trained on population activity evoked by the entire stimulus display during the attention period? To answer this, we examined the decodable information during the delay epoch in the different subpopulations of neurons and in the entire population (Fig. 10). We trained linear classifiers using firing rates from ipsilateral-tuned units, contralateral-tuned units, and the full population, trained and tested within short-duration windows over the duration of the delay epoch (stepped by 25 ms; see Materials and Methods). First, we note that the classification accuracy exceeded 95% on average, despite using very small integration time windows of 25 ms (Fig. 10*A*, black curve). Secondly, classification accuracies for to each oppositely-tuned population (contralateral: orange; ipsilateral: blue) both decreased following distracter onset, with the magnitude of decrease being greater in the ipsilateral-tuned population than contralateral-tuned population.

Note that the decoding accuracy decreased to a level that was still well above chance, suggesting that even when distracters caused the firing rates to evolve toward those evoked by the four stimuli during fixation (Fig. 8), the population code still signals the target location. A possible explanation for this result is that the classifier may use information from a subpopulation of neurons (e.g., contralateral neurons) that are less affected by the appearance of the distracter (orange curve). This suggests that resilience to distracter interference may be a feature of this subpopulation of contralateral neurons. Here one may consider that classifiers using single neurons as features do not use central tendency statistics such as mean differences across the population (as does the state space analyses illustrated in Fig. 8) but can use information from only a subset of neurons and perform well above chance. Indeed, we found that both populations converged toward the same level of classification accuracy, despite there being more contralateral neurons relative to ipsilateral neurons, and despite attentional effects being on average larger in contralateral neurons.

Several conclusions can be derived from these results. First, that a linear classifier would approach ideal performance when using the population code based on the activity profile evoked by both targets and distracters compared to when using a code based on activity evoked by the target alone (Fig. 9). This can be further illustrated by the confusion matrices shown in Figure 10*B–D*. The matrix for the full population shows a yellow diagonal illustrating the classifier made almost no errors, while the other matrices diagonal show shades of orange (see color scale). Second, that each subpopulation alone reached a lower performance than the entire population considered together. Finally, that the activity of a relatively small population of area 8a neurons (*n* = 232) is sufficient to encode the allocation of attention to one of four target locations in the presence of distracters using a rate code that integrates information during time intervals as short as 25 ms.

## Discussion

We found that responses of neurons in LPFC area 8A to multiple stimuli across the visual field were normalized, and that the magnitude of normalization of a neuron’s response was related to its visuospatial tuning: the responses of ipsilateral neurons were more strongly normalized than the responses of contralateral neurons. Attention affects contralateral and ipsilateral neurons responses by shifting the normalization computations from AVG to WTA, an effect that was significantly stronger in contralateral neurons. We reduced the dimensionality of the data set by pooling the responses of all contralateral and all ipsilateral neurons and analyzed the temporal dynamics of neural activity. Neural trajectories for the different trial types departed from a common point, diverged during the cue period, converged during distracter presentation (AVG), and diverged again during the allocation of attention (WTA). Finally, we used linear classifiers to decode the locus of covert spatial attention from LPFC neuronal activity. Classifiers were able to decode the allocation of attention on a single-trial basis at significantly above chance levels when using a rate code derived from neuronal responses to a single target stimulus earlier in the trial. When using a code based on neural responses to the target and distracters during the period of sustained attention, classification accuracy substantially improved, approaching ideal performance with a temporal resolution as high as 25 ms.

### Response normalization in LPFC area 8a neurons

Response normalization can be described as a canonical computation in which the response of a neuron is divided by a common factor that typically includes the summed responses of a pool of neurons (i.e., a normalization pool (Heeger, 1992; Carandini and Heeger, 2012). We found 232/236 (98%) of recorded neurons in LPFC area 8A exhibited sublinear responses to multiple stimuli in their RF, compatible with normalization, and were best characterized by an average (AVG) computation. One possible limitation to our study is that our quantification of normalization relied on comparing responses of individually-presented stimuli to those of multiple stimuli presented simultaneously. Specifically, it is important to note that responses to individual stimuli may be affected by task demands since the animals knew to attend toward the stimulus during the subsequent delay period. However, similar findings have been reported across multiple visual cortical areas (Recanzone et al., 1997; Britten and Heuer, 1999; Gawne and Martin, 2002; Lampl, 2004; Zoccolan et al., 2005; but see Finn and Ferster, 2007; Maunsell, 2015). Normalization allows a neuron to encode responses to multiple stimuli in the visual field using a similar dynamic range, independently of the number of presented stimuli (Carandini and Heeger, 2012). As the LPFC signals the allocation of top-down attention to a target among distracters, it may be important that neurons in this area maintain an appropriate bandwidth, calibrating neuronal responses to variable numbers of stimuli across the entire visual field before allocating attention.

One distinctive feature of area 8a neurons is the large size of their RFs, which may span both ipsilateral and contralateral hemifields (Mikami et al., 1982; Funahashi and Bruce, 1989; Bullock et al., 2017). Indeed, we found neurons with RFs spanning up to all four quadrants of the visual field (Fig. 4). Neurons with larger inhibitory RFs (RFi) underwent stronger response normalization relative to neurons with smaller RFi. Many of the strongly normalized neurons exhibited preference for the ipsilateral visual hemifield, suggesting that these neurons have stronger inhibitory drive than contralateral neurons. Importantly, the large size of excitatory and inhibitory RFs of these LPFC neurons allows the calibration of responses to multiple stimuli across the entire visual field. This becomes important for signaling the allocation of attention across the entire visual field and contrasts with neurons in visual cortex, in which RFs rarely extend across both visual hemifields.

A recent study mapping visually responsive neuron RFs in area 8a showed that ipsilateral neurons possess larger RFi than contralateral neurons (Bullock et al., 2017). In agreement with this result, we found that ipsilateral neurons underwent stronger normalization than contralateral neurons when presented with multiple stimuli across the visual field (Figs. 3, 4). One possible explanation for this result may be linked to the finding that contralateral neurons comprise ∼70% of the population in the area compared to ∼30% ipsilateral neurons (Lennert and Martinez-Trujillo, 2013; Bullock et al., 2017; Fig. 3). If we assume that (1) contralateral and ipsilateral neuronal populations are mutually inhibitory, and (2) that the strength of the inhibition is proportional to the size of the neuronal pool, then the average ipsilateral neuron would receive more inhibition than the average contralateral neuron. Thus, in response to multiple stimuli spanning the entire visual field, the inhibitory drive to the ipsilateral population would be larger than the one to the contralateral population. This would result in stronger response normalization in ipsilateral neurons when multiple stimuli are similarly distributed across both hemifields. Although we recorded from the left LPFC, a similar predominance of neurons tuned for the contralateral visual hemifield has been reported in the right LPFC of macaques (Lennert and Martinez-Trujillo, 2013), suggesting that this asymmetry in pool size is a feature of the LPFC independent of its hemispheric location. The functional role of this difference in normalization strength between populations is unclear.

### Attentional modulation across neuronal subpopulations

The effects of attention on the responses of ipsilateral and contralateral LPFC neurons have been previously reported (Lennert and Martinez-Trujillo, 2013). In general, attending to stimuli inside the RF increased the activity of both tuned subpopulations relative to attending outside. However, this attentional modulation is stronger in contralateral neurons, which agrees with previous reports (Lennert and Martinez-Trujillo, 2013). We elaborate on this finding, showing that with attention, responses of contralateral neurons resemble a WTA computation while responses of ipsilateral neurons remain better characterized by an AVG computation (Fig. 6). These differences in attentional modulation may be linked to the differences in normalization we found between the two populations of neurons.

The link between normalization and attention has been proposed by several studies (Reynolds and Heeger, 2009). Some studies have proposed that attention increases the gain of excitatory inputs corresponding to the attended stimulus before normalization (Ghose, 2009; Lee and Maunsell, 2009; Reynolds and Heeger, 2009). This attention-normalization framework is built on the assumption that a single neuron undergoes at least two stages of computation when converting synaptic inputs into trains of action potentials. Neurons first integrate tuned inputs from upstream neurons, and in the second stage this tuned signal is weighted or normalized by the responses of neighboring neurons via inhibitory connections (Carandini and Heeger, 2012). To expand on the link between response normalization and our results, we will first consider the normalization equation proposed by Reynolds and Heeger (2009) in a simplified form:

response=E/[E+I]

where *response* is the neuron’s response, E is the excitatory tuned input from upstream neurons and I is the additional inhibitory input from the normalization pool. For the population of contralateral neurons, the equation can be written as:

$${\mathrm{response}}_{c\mathrm{ontra}}=E_{\mathrm{contra}}/[E_{\mathrm{contra}}+E_{\mathrm{ipsi}}]$$

where $E_{\mathrm{contra}}$ represents the integral of the number of spikes per time unit (firing rate) of the contralateral population and $E_{\mathrm{ipsi}}$ the same quantity but corresponding to the ipsilateral population. Similarly, the equation for the ipsilateral population can be written as:

$${\mathrm{response}}_{\mathrm{ipsi}}=E_{\mathrm{ipsi}}/[E_{\mathrm{ipsi}}+E_{\mathrm{contra}}]$$

We can then apply the same attentional gain *G* to one or the other population depending on where attention is allocated (i.e., to the hemifield contralateral or ipsilateral to the recording site):

$${\mathrm{response}}_{\mathrm{att}-\mathrm{contra}}={G*E}_{\mathrm{contra}}/[G*E_{\mathrm{contra}}+E_{\mathrm{ipsi}}]$$

$${\mathrm{response}}_{\mathrm{att}-\mathrm{ipsi}}={G*E}_{\mathrm{ipsi}}/[G*E_{\mathrm{ipsi}}+E_{\mathrm{contra}}]$$

where ${\mathrm{response}}_{\mathrm{att}-\mathrm{contra}}$ and ${\mathrm{response}}_{\mathrm{att}-\mathrm{ipsi}}$ are the responses of the two populations with attention. Because $E_{\mathrm{contra}}>E_{\mathrm{ipsi}}$ (due to the asymmetric population sizes), when applying the term *G*, the response increase in the contralateral population will be proportionally larger than in the ipsilateral population. Thus, without changes in the strength of the signal that triggers the attentional modulation (*G*), the asymmetries in the size of the populations can result in a greater increase in firing rates in contralateral than in ipsilateral neurons and thus a departure from the AVG computation toward a WTA in the contralateral population.

Although our hypothesis needs to be further tested, some findings from previous studies seem to provide some support its favor. First, it has been shown that callosal projections from ipsilateral parietal cortex and contralateral prefrontal cortex interdigitate in the LPFC (Goldman-Rakic and Schwartz, 1982; Goldman-Rakic et al., 1984). These anatomic observations likely correspond to the segregation of ipsilateral and contralateral neurons observed in our MEA (Fig. 5). Second, it has been shown that ipsilateral and contralateral neurons show differences in their response latencies and it has been proposed that they may play different roles in the generation of saccades and anti-saccades (Johnston et al., 2009). In the same study the authors showed that a relatively high proportion of neurons possessing narrow waveforms were ipsilateral-tuned, and further suggested that they may be inhibitory interneurons that target contralateral neurons within the same hemisphere of LPFC. It has been shown that contralateral and ipsilateral LPFC neurons show a higher proportion of negative spike count correlations, which may indicate inhibitory interactions between them (Leavitt et al., 2013, 2017). Overall, these results support the hypothesis that during both response normalization and the allocation of attention, ipsilateral and contralateral neurons engage in competitive interactions through lateral connections. Since this process is likely to occur in both hemispheres, it leads to the questions of what the function is of such a redundant system, and whether and how computations from both hemispheres are combined into a single saliency map.

### Temporal dynamics of attentional modulation

Temporal dynamics of activity in ipsilateral and contralateral LPFC neurons have been shown to differ (Lennert and Martinez-Trujillo, 2013). Here, we additionally found that ipsilateral neuronal activity in response to a single stimulus was suppressed by the distracter appearance more rapidly than that of contralateral neurons. This may be related to the stronger impact of normalization on ipsilateral neurons. Interestingly, following distracter onset, both populations tended toward a WTA state at similar rates while attention was sustained on the target. These dynamics may be explained within the normalization framework; normalization models capture the bottom-up distracter response, as in the responses during fixation trials, with ipsilateral neurons undergoing stronger and more prolonged normalization. The asymmetry in the strength of inhibitory inputs (see previous section) may result in the greater magnitude and faster dynamics of normalization in ipsilateral neurons. Importantly, a similar attentional gain factor *G* applied to the responses of each population when attention is directed to the contralateral or ipsilateral hemifield increases the firing rate of the corresponding population at a similar rate. However, because the ipsilateral population undergoes greater normalization due to distracter appearance, the amount of increase and therefore similarity to a WTA regime is smaller in the ipsilateral population. Thus, the differences in temporal dynamics can be explained by differences in the strength of normalization in the different subpopulations, rather than by difference in the strength of the attentional signal bias, represented by *G*. The origins of *G* are not clear, but they may originate from working memory representations of spatial locations activated by the cue, or by a persistent sensory representation of the cue (Mendoza-Halliday and Martinez-Trujillo, 2017).

One potential pitfall of our state space analysis is that the dimensionality reduction is based on the spatial preferences of the neurons. Some prior studies have opted for alternative approaches such as principal component analyses to reduce the dimensionality of the dataset and plot the trajectories as a function of trial time in state space (Murray et al., 2017). Our approach decomposes the neuronal activity based on experimenter-defined task-relevant dimensions, and thus we consider it to fall in the category of “targeted” dimensionality reduction, similar to other prior work in LPFC (Mante et al., 2013) and FEF (Ebitz et al., 2018). Our approach may also be justified based on the distinction between the response properties of the ipsilateral and contralateral neurons and their anatomic segregation within the LPFC (Goldman-Rakic and Schwartz, 1982; Lennert and Martinez-Trujillo, 2013). It is plausible that downstream projections from contralateral and ipsilateral neurons reach similar downstream neurons or populations that integrate information across the entire visual field. The trajectories plotted in Figure 8 remain segregated for the different allocations of attention suggesting that the activity of the two subpopulations could convey information to downstream mechanisms. This information can be used to generate motor plans, e.g., saccades with different directions to different possible target locations during the response period, or to feedback into visual areas to modulate the processing of sensory signals.

The concept of segregated ipsilateral and contralateral coding may also be justified by the impact that the meridians of the visual field exert on attention and working memory, both behaviorally (Carlson et al., 2007; Liu et al., 2009; Alvarez et al., 2012) and at the neuronal level in LPFC (Buschman et al., 2011; Matsushima and Tanaka, 2014; Leavitt et al., 2018). Strong inhibitory interactions between contralateral and ipsilateral neurons may also explain why distracter interference is lower during attention tasks where targets and distracters are located in either the opposite or same visual hemifield (Stormer et al., 2014). These inhibitory interactions may facilitate implementation of WTA strategies between targets and distracters in different hemifields. Future studies using techniques that allow identification of neuronal types in behaving animals are needed to reveal the details of the microcircuitry underlying these interactions.

Wimmer et al. (2016) recorded from PFC neurons while macaques performed a delayed compare-to-sample task with random dot patterns. It is difficult to draw major conclusions between our results and theirs because we compared the properties of two classes of neurons (neurons preferring ipsi vs contra stimuli), whereas they compared the properties of two classes of neural responses (responses to ipsi vs contra stimulus presentations). Nevertheless, there are some comparisons that can be made. Both of our studies found a higher proportion of excitatory than inhibitory neurons, although we observed approximately a 60:40 ratio of excitatory to inhibitory neurons whereas they reported a 79:21 ratio. Wimmer et al. (2016) found that stimulus selectivity, as measured using auROC, was similar for contralateral and ipsilateral stimuli. Likewise, our population decoding analysis showed that the amount of contralateral and ipsilateral stimulus information is similar during the latter portion of the attentional delay for a classifier trained and tested on the same time bin (Fig. 10). However, we also observed that dynamics in the population representation diminish the information about ipsilateral stimuli across the cue and early delay epochs (Fig. 9).

### Ensemble coding of attention

The translation from single neuron responses to ensemble coding and information read-out is nontrivial (Moreno-Bote et al., 2014). However, using linear classifiers allows us to quantify the evolution of the population state similarity relative to when the attended stimulus is presented alone (i.e., ideal WTA). A study using similar methods to these found that attentional modulation drives the responses of inferotemporal neurons toward a WTA state (Zhang et al., 2011). We found that a classifier trained on neuronal firing rates during the cue epoch could be used to reliably decode the location of covert spatial attention during the delay epoch (Fig. 9). This shows that the population response to a stimulus presented in isolation explains a substantial portion of the population response variance when attending to that stimulus in the presence of distracters. This finding is consistent with attentional modulation resembling a WTA response, and agrees with models in which attention biases neuronal population activity toward a state resembling that when the attended stimulus is presented in isolation (Desimone and Duncan, 1995).

On distracter onset, neurons that were previously silent became transiently excited as stimuli populated their RFs; concurrently, neurons that were previously excited underwent normalization (suppressed response) when additional stimuli appeared. The effect of this was akin to a decrease in signal-to-noise. As a result, classification accuracy of cue epoch-trained decoders tested on stepped time windows of firing rates during the delay epoch steeply declined. However, we found that sustained attentional modulation resulted in a steadily increasing classification accuracy as time progressed. This indicates that the population state dynamically migrates toward a state resembling isolated cue stimulus presentation (Zhang et al., 2011). The classifier was more prone to making errors on trials when the monkey was allocating attention to the ipsilateral hemifield. This was likely due to contralateral neuronal activity reaching a state closer to an ideal WTA computation while ipsilateral neurons, although significantly modulated during attend in trials, remained best described by AVG. In general, at the full ensemble level, attentional modulation did not result in a full WTA computation resembling the activity profile evoked by the single cue, as the classifier accuracy plateaued at performance levels below 100%.

Finally, we used linear classifiers trained and tested on the attentional delay epoch firing rates (Fig. 10). This contrasts with our previous analysis using classifiers trained on the cue epoch when the attended stimulus was presented in isolation. By training on the delay epoch, we are assuming that a downstream reader is not using the template provided by the single cue alone (WTA template). In this scenario, the downstream reader would use the template corresponding to the population response that underwent response normalization and partially migrated to a WTA regime with attention. We found that the read-out of information from an exclusively ipsilateral-tuned ensemble was not different from that of an ensemble composed of exclusively contralateral-tuned neurons. This finding remained true when controlling for neuron receptive field size, as well as population size. Thus, despite ipsilateral-tuned neurons not computing a WTA, as contralateral neurons did, they encoded sufficient information regarding the allocation of attention through changes in response profiles.

Decoders using the full ensemble (232 neurons) activity during the attention period achieved near-perfect classification accuracy after distracter onset (although this was dependent on window size used to compute firing rates; see Materials and Methods). If we use classification accuracy as a proxy for information content in the ensemble (Moreno-Bote et al., 2014; Tremblay et al., 2015; Boulay et al., 2016; Leavitt et al., 2017), we can conclude that a downstream neuron reading out normalized activity performs better than one that assumes a WTA population response. This is not surprising when considering that monkeys as well as all other animals rarely encounter environments in which targets exist in isolation, but environments in which targets coexist with multiple distracters. Under this assumption, neuronal activity should always be normalized in cortical circuits, so normalization is the norm and not the exception. Thus, the weights of the classifiers trained during the target and distracters (delay) period are a more realistic estimation of the weights corresponding to read out downstream mechanisms in natural conditions.

## Conclusions

**Figure 11.**
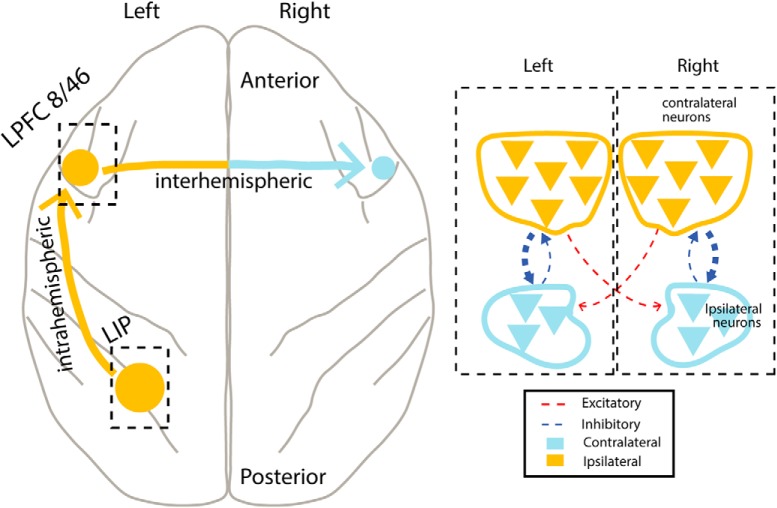
Hypothetical normalization circuit in the LPFC. The diagram illustrates a top view of the left and right hemispheres of a macaque monkey brain. The arrows illustrate the origin of excitatory inputs into the pools of contralateral and ipsilateral neurons. The arrows illustrate excitatory (red) or inhibitory (blue) connections between pools of neurons. Triangles are individual units. The colors indicate their spatial preference.

We characterized the properties of LPFC area 8A neurons during different periods of a task that required sensory processing of a single stimulus (the cue) and filtering out of distracters appearing across the visual field. We described two groups of neurons, ipsilateral and contralateral tuned, that show differences in their response properties regarding normalization and attention. These two groups of neurons likely receive inputs from different brain areas and compete through inhibitory interactions (see diagram in Fig. 11) to produce a saliency map at the level of the population that can be read out by a downstream mechanism to accurately signal the allocation of attention. More generally, our results provide evidence in favor of the existence of a circuit mechanism in primate LPFC that flexibly operates across the entire visual field and dynamically performs different normalization computations (AVG and WTA) to generate signal maps of behavioral relevance of the entire visual field. This normalization circuit may have evolved in primates with the expansion of the LPFC to allow cognitive flexibility.

## Synthesis

Reviewing Editor: Tatiana Pasternak, University of Rochester Decisions are customarily a result of the Reviewing Editor and the peer reviewers coming together and discussing their recommendations until a consensus is reached. When revisions are invited, a fact-based synthesis statement explaining their decision and outlining what is needed to prepare a revision will be listed below. The following reviewer(s) agreed to reveal their identity: Bruno Averbeck.

The paper provides a compelling demonstration that LPFC activity during a cued change detection task is stronger for stimuli presented in the contralateral than in ipsilateral hemifields or for unattended stimuli. The novelty of the work lies in the use of multiple stimuli, a study of attentional modulation of responses to these stimuli and in the observed response normalization. While generally positive, the reviewers raised several issues that must be addressed before the paper can be accepted for publication in eNeuro. These are summarized below and should be addressed in the revised manuscript.

1. Both reviewers felt the task description is missing important details. Please clarify the task, provide stimulus details and respond to specific questions in the reviews below (see Reviewer 1, points 3-5, Reviewer 2, points 1-2)

2. Address the point about excitatory and inhibitory responses raised by Reviewer 1 concerning averaging the two types of responses.

3. Include and discuss the missing relevant references as suggested by Reviewer 1 (see “8”).

4. Address questions from Reviewer 2 concerning your approach to dimensionality reduction analysis and about cross-validation of the decoding analysis

5. Please correct other minor problems listed in the two reviews.

REVIEWER #1

Advances in the field:

Response normalization has previously be studied mainly in sensory cortices (e.g. V4, MT) and this report now studies normalization in PFC, an area that is thought to provide top-down attentional signals to sensory cortices. The advance concerns the representation of multiple stimuli in PFC and the mechanisms of attentional modulation.

Comments to the authors:

Response normalization has previously be studied mainly in sensory cortices (e.g. V4, MT) and this report now studies normalization in PFC, an area that is thought to provide top-down attentional signals to sensory cortices. The advance concerns the representation of multiple stimuli in PFC and the mechanisms of attentional modulation.

The manuscript investigates the representation of visual stimuli (Gabor gratings) in firing rates of neurons in lateral prefrontal cortex of macaques during a spatial attention task. The first half of the paper concerns the receptive field structure of PFC neurons, measured from responses to stimuli in one or four quadrants of the visual field. Consistent with previous reports, many neurons have large receptive fields and respond to stimuli in ipsi- and contralateral hemifields. Neural responses to four stimuli were approximately equal to the average response to each of thes four stimuli presented in isolation. The second half of the paper investigates the impact of attention on this normalization effect. Results include a difference in normalization of neurons that prefer ipsi- vs contralateral stimuli giving hints about the potential underlying circuitry.

Overall, the paper is well written and contains interesting findings that are well-suited for publication in eNeuro. I think the paper covers a lot of material, some of which is more exciting than other (for example, there seems to be significant anatomical clustering just in one of the two monkeys and a whole figure is devoted to this). Nevertheless, I think that all the results are worth to be reported even if they make the paper longer and sometimes not that focused.

I had some troubles with the description of the task but assuming that I interpreted the paper correctly, my concerns are all rather minor.

1. “inhibitory” responses vs “exc” responses: the fact that responses can be inhibitory for all spatial locations (response below baseline) is introduced in Fig 4. I would suggest to plot the z-scored firing rate in Fig 3B separately for the different neuron types in order to illustrate this. In general, averaging across suppressive and excitatory neurons gives smaller responses an seems not appropriate. In the same line: how are preferred locations defined for suppressive neurons? As the minimum response across all possible four locations?

2. Stimuli: Information about the size of the stimuli, and other stimulus attributes is missing (spatial frequency of the Gabors, speed, etc).

3. Task description is not complete. For example: What is the percentage of target, distractor, and target+distracter trials? How where the target+distracter stimuli chosen (always opposite?) When is the monkey rewarded? What is the condition to get a reward in the fixation trials? Please add the missing info and revise the methods.

4. Mean success rate: how is the success rate defined for fixation trials? Success rate in fixation trials is &lt; 80%. Does this mean that fixation broken in ~20% of the trials? How often is fixation broken in the attention trials?

5. Why is chance level 25% (line 300)? As I understand, chance level is not just given by selecting one out of four targets, but by the fraction of the different trial types (it is not a forced choice task).

6. Line 357: should it be “This difference” as there is just one difference between ipsi and contra?

7. line 364: median is not very informative here because there are just 4 different possible vales; give mean in addition.

8. Relevant literature. Several papers from the Pasternak lab have investigated the responses of PFC neurons to visual (motion) stimuli in detail, yet they are not cited. In particular, the recent paper Wimmer et al 2016, J. Neurosci. DOI:10.1523/JNEUROSCI.0843-16.2016 seems relevant as it contains results about contra- and ipsi-lateral response strength in PFC as well as a report of excitatory and suppressive responses and their relative incidence. I suggest to compare / contrast the current findings with this previous paper and other previous findings where appropriate.

9. Attention responses in Fig 6A: is it possible to show the responses starting from stimulus onset rather than distracter onset? This would reveal the build-up of the attentional modulation and the magnitude of the response (decrease compared to maximum response as in the avg model or constant as for the WTA model).

10. Fig 9B: It is interesting that decoding is worse for ipsilateral stimuli. It is stated that this is not due to fewer ipsi-preferring neurons. Have you tested whether the overall response strength (response amplitude) is lower in ipsi-neurons than in contra-neurons? Perhaps this could explain the difference?

REVIEWER #2

Advances in the field:

This manuscript shows that lateral prefrontal activity reflects the average response to a set of cues when arrays are not attended, or when they are attended and the cells are tuned for ipsilateral locations. However, when cells are tuned for contralateral locations activity reflects the best cue.

Comments to the authors:

The authors describe a study in which they record from neural activity in lateral PFC while animals carry out a cued change detection task. They look at whether neural responses when multiple stimuli are presented, and are either attended or not, look like the responses to single stimuli. They compare these using sum, average and max response models.

Overall this paper makes a compelling case for response normalization in unattended conditions in PFC. When attention is directed into the response field of a neuron, neurons that code for contralateral space respond as if that was the only cue present. However, in other conditions the response is closer to the average. I have some comments which, if addressed, could increase the clarity of the study.

1. A few details of the task were not clear. Not sure if I missed them somewhere. I can make them out from the figure, but it would be useful to have them written in the methods. In the distracter condition, I guess the animal is supposed to withhold its saccade. When the animals did make errors in this condition (50%) did they saccade to the rotated target? So they had forgotten the location of the cue? Where the cue locations blocked or did they change every trial? Seems they changed, but this should be made clear.

2. Were the gratings always the same orientation? Is it assumed that cells are not strongly tuned to orientation?

3. In several locations it says “reference to be included” but no reference was included.

4. Comparisons between the cue trials of the attend condition and delay activity of the fixation condition also have a task confound. The task requirements may affect the responses of some cells. This could just be briefly mentioned in the discussion.

5. On line 182 in the methods there is a reference to a variable n, but it does not occur in any equations.

6. For the dimensionality reduction analysis, was the average activity at each point in time used, or was one average used per cell across time? In other words, you could project onto the PSTH, or you could project onto a grand mean. Which was done?

7. Was the decoding analysis cross-validated? When the regularized logistic regression was used?
